# Clinical and radiographic outcomes of mini-implant-retained maxillary and mandibular overdentures: a systematic review and meta-analysis

**DOI:** 10.1007/s00784-025-06242-3

**Published:** 2025-03-01

**Authors:** Morteza Mohammadi, Emad Baker, Bruno Ramos Chrcanovic

**Affiliations:** 1 Faculty of Odontology, Malmö University, Malmö, Sweden; 2 Department of Oral and Maxillofacial Surgery and Oral Medicine, Faculty of Odontology, Malmö University, Carl Gustafs väg 34, Malmö, SE-214 21 Sweden

**Keywords:** Mini-implant, Overdenture, Failure, Marginal bone loss, Technical complications, Systematic review, Meta-analysis

## Abstract

**Objective:**

To assess the clinical and radiographic outcomes of overdentures and their retaining dental mini-implants, based on a single-arm systematic literature review.

**Methods:**

Electronic search was undertaken in three databases, last updated in October 2023, plus manual search of journals. Cumulative implant survival rate (CSR) and the estimated marginal bone loss (MBL) under different follow-up times were calculated.

**Results:**

Thirty-nine studies were included, with 3,787 mini-implants supporting 1,026 overdentures (109 maxilla, 896 mandible) in 1,005 patients, with a mean follow-up of 28.1 ± 19.8 months (min-max 0.3–84.0). 202 mini-implants failed, after a mean of 9.4 ± 11.8 months (7-year CSR 91.4%). The implant survival was lower in the maxilla in comparison to the mandible (*p* < 0.001), lower for early/delayed-loaded in comparison to immediately-loaded implants (*p* = 0.005) and lower for 2-mini-implant in comparison to 4-mini-implant-retained mandibular overdenture (*p* < 0.001; Log-rank test). A high rate of transversal fracture of the prosthesis and wear of the attachment parts was observed. The estimated mean MBL gradually increased from 0.518 (≤ 6 months) to 1.260 mm (58.8–90 months). There was an estimated MBL increase of 0.011 mm per additional month of follow-up.

**Conclusion:**

Although presenting a high 7-year CSR, mini-implant-retained overdentures may require frequent maintenance follow-ups, due to the high rate of technical complications. The estimated MBL of mini-implants over 80 months is low.

**Clinical relevance:**

The ability to anticipate outcomes is an essential part of risk management in clinical practice. The findings reported herein set some recommendations and potential strategies for minimizing failures and complications commonly associated with this mini-implant-retained overdentures.

**Supplementary Information:**

The online version contains supplementary material available at 10.1007/s00784-025-06242-3.

## Introduction

Most countries in the world are presenting growth in the proportion of older persons in their population. According to the latest United Nations population estimates and projections, one in six people in the world will be over age 65 (16.4%) by 2050, up from one in 11 in 2019 (9.7%) [1]. And the older one gets, the higher the risk of edentulism. Although the rates of edentulism have been slowly falling in some countries [2], the prevalence of edentulism is still very high among the elderly, as these are at greater risk for poor oral health and tooth loss as compared with those < 65 years of age, with dental caries and periodontal disease being the main causes of tooth loss [3]. Recent estimated on the global prevalence of edentulism among individuals aged 45 or older is 22% [4].

With the edentulism comes the resorption of the alveolar socket and volume decrease of the jaw bone [5, 6], a process that continues even after patients begin to wear removable complete dentures [7], all of which can significantly alter the shape of the jaws [8].

The lack of enough bone volume for the placement of traditional, normal-diameter implants in the edentulous jaws leaves some options for oral prosthetic rehabilitation. One would be the removable complete denture, that lies directly on the gingiva/mucosa of the edentulous alveolar ridge, which is a cheap, straightforward process to manufacture, and easy to maintain by the patient, but which can bring discomfort, become loose, and the patient may have problems to adapt to it [9]. The edentulous patient’s chewing capacity and bite force remain both impaired, as this type of prosthesis may become unstable or lack retention due to poor availability of residual bone [10]. Moreover, removable complete dentures can give rise to lesions of the oral mucosa [11].

Another option would be to graft the alveolar ridge with additional bone to create sufficient bone volume for the placement of implants of conventional diameter [12, 13]. However, these interventions are invasive and subject to intraoperative and postoperative complications, such as exposure of the grafted material, infection, neurosensorial disturbances, hemorrhage, pain, among others [14], besides being costly for the patient.

The use of mini-implants would be a third alternative. Mini-implants consist of a dental implant that is fabricated with a reduced diameter, up to 3.0 mm [15], while narrow or conventional diameter implants have a diameter greater than 3.0 mm. As advantages, the placement of mini-implants is simpler and less invasive, and the entire procedure is also less time-consuming in comparison to the installation of implants of wider diameter [16]. Moreover, due to their narrow diameter, mini-implants can be placed in jaw sites with less bone volume and less thickness, minimizing the need for bone grafting procedures in atrophic ridges. Furthermore, the cost of mini-implants is significantly less than implants with wider diameters [17]. In relation to removable complete denture, patients rehabilitated with overdentures retained by mini-implants are more satisfied, due to increased retention and stability of the prosthesis, increased mastication efficiency, improved comfort, and security in social life [10, 18, 19].

However, there are potential problems and complications associated with the use of mini-implants to support overdentures. These implants present a higher risk of fracture, due to its narrow diameter [20], as well as due a less-than-ideal balanced distribution of the forces of mastication in this type of prosthesis, leading to a high stress concentration on implants that are nearest to the place of loading [21]. Moreover, narrow-diameter implants may be associated with greater marginal bone loss in comparison to standard-diameter implants [22]. In addition, elderly individuals, who are more prevalently rehabilitated with implant-supported overdentures, usually present an increase in cognitive and physical disabilities, which can lead to poor oral hygiene [23]. Poor oral hygiene is suggested to be a risk for peri-implant health, leading to chronic inflammation and ultimately to loss of implants [24]. Other systematic reviews separately assessed the clinical outcomes of mini-implant-retained overdentures in the maxilla and in the mandible. These reviews reported a lower survival rate for mini-implants in the maxillary cases (77.1%, mean follow-up of 1.79 years) [25] than the mandibular ones (95.6%, mean follow-up time of 28.2 months) [26]. However, the authors of these reviews reported plain survival rates, without properly looking into the time factor. Moreover, the radiographic outcomes were not assessed.

The purpose of the present study was to assess the clinical and radiographic outcomes of maxillary and mandibular overdentures and their retaining dental mini-implants, based on a systematic review of the literature.

## Materials and methods

This single-arm systematic review followed the PRISMA Statement guidelines [27]. The review was registered in PROSPERO (CRD42023487478).

### Research question

The focused question was: What is the failure rate, the prevalence of technical complications, and the estimated mean marginal bone loss of dental mini-implants and mini-implant-retained overdentures used for the rehabilitation of patients with edentulous jaws?

### Search strategies

An electronic search without time restrictions was firstly undertaken in August 2022, last updated in October 2023, in the following databases: PubMed/Medline, Web of Science, and Scopus. The following terms were used in the search strategies:

(“mini-implant” OR “mini dental implant” OR “small diameter dental implant” OR “small diameter implant” OR “small dental implant” OR “narrow diameter dental implant” OR “narrow diameter implant” OR “narrow dental implant” OR “provisional implant” OR “temporary implant”) AND (overdenture OR “coping prosthesis” OR “overlay prosthesis” OR “overlay denture” OR “full denture” OR denture OR “complete denture”).

A manual search of the following journals was performed: *Clinical Implant Dentistry and Related Research*,* Clinical Oral Implants Research*,* European Journal of Oral Implantology*,* Implant Dentistry*,* International Journal of Implant Dentistry*,* International Journal of Oral and Maxillofacial Implants*,* International Journal of Oral Implantology*,* International Journal of Prosthodontics*,* Journal of Clinical Periodontology*,* Journal of Oral Implantology*,* Journal of Periodontology*,* Journal of Prosthetic Dentistry*,* Journal of Prosthodontics*,* Journal of Prosthodontic Research*. The reference list of the identified studies and the relevant reviews on the subject were also checked for possible additional studies.

### Inclusion and exclusion criteria

Eligibility criteria included clinical human studies, either randomized or not, interventional or observational, reporting case series of patients rehabilitated with mini-implant-retained overdenture. Case reports were also considered, provided that follow-up information was reported. Implants of titanium (c.p.Ti) or its alloys were included.

The following cases were excluded: (1) patients receiving overdentures retained/supported simultaneously by teeth and implants; (2) case series report from which no individual patient data could be extracted; (3) partial overdentures; (4) cases with no prosthetic follow-up– cases with follow-up time only between the implant placement and prosthesis installation were not considered as the main aim of the present review was to evaluate the prosthetic phase; (5) studies suspected to present duplicated cases, usually originating from clinical series from the same service or university, but in different articles/publications; (6) case series studies with shorter follow-up time will not be considered, provided that a longer follow-up publication will be available; (7) case series of patients treated for head and neck cancers and/or ablative surgery of the jaws, as these cases are already expected to present an increased failure rate of implants [28], and also due to the fact that the clinical outcomes for these cases could strongly deviate from patients not in the same situation, due to, for example, osteoradionecrosis, constant ulceration of the oral mucosa, xerostomia, among others [29, 30].

### Study selection

The titles and abstracts of all reports identified through the electronic searches were read independently by the authors. For studies appearing to meet the inclusion criteria, or for which there were insufficient data in the title and abstract to make a clear decision, the full report was obtained. Disagreements were solved by discussion between the authors.

RefWorks Reference Management Software (Ex Libris, Jerusalem, Israel) was used in order to detect duplicate references in different electronic databases.

### Quality assessment

Quality assessment was executed according to the Quality Assessment Tool of the National Institutes of Health [31]. The NIH quality assessment tool calculates the study quality on the basis of nine criteria. The ratings on the different items were used by the reviewers to assess the risk of bias in the study due to flaws in study design or implementation. The studies were classified as “good,” “fair,” or “poor” quality. In general terms, a “good” study has the least risk of bias, and results are considered to be valid. A “fair” study is susceptible to some bias deemed not sufficient to invalidate its results. The fair quality category is likely to be broad, so studies with this rating will vary in their strengths and weaknesses. A “poor” rating indicates significant risk of bias. Studies of “good” quality were judged to have at least 7 points.

Quality assessment of the included case report publications was carried out according to the 13-item CARE guidelines [32] were used for the quality assessment of the case report articles. A score of 1 was given for each item outlined in the CARE guidelines, with a maximum score of 30 for a case report. A score of 30 represents the highest quality, and two-thirds or more of the points are considered high quality.

The reviewers went together through all the items of the NIH quality assessment tool for five case-series studies as an initial calibration, after which the quality assessment was carried out independently by the reviewers. Any disagreement was resolved by discussion between the authors. All the reviewers performed together the quality assessment of the case report publications, due to the small number of included publications of this type.

### Definitions

A mini-implant was defined as a dental implant that is fabricated with a reduced diameter (up to 3.0 mm) with the same biocompatible material as compared with standard dental implants [15].

An implant was considered a failure if presenting signs and symptoms that led to implant removal, i.e., a lost implant. Implant failure could be either early (the inadequacy of the host to establish or promote osseointegration in the early stages of healing) or late (the failure of either the established osseointegration or function of dental implants) [33]. Fracture of an implant was also considered as a failure [20].

A mini-implant-retained overdenture was defined any removable dental prosthesis that covers and rests on one or more dental mini-implants.

An overdenture was considered a failure in the following situations: (1) loss of the supporting implant(s); (2) new implants were placed in the jaw in order to support a fixed full-arch prosthesis; (3) change of attachment system; and (4) complete transverse fracture of the overdenture.

MBL was defined as loss, in an apical direction, of alveolar bone marginally adjacent to the dental implant, in relation to the marginal bone level initially detected after the implant was surgically placed [34]. Studies using the long-cone parallel technique for periapical radiographs were considered.

### Data extraction

From the studies included in the final analysis, the following data was extracted: number of patients, patients’ age and sex, implant healing period, number of supporting implants per overdenture, implant surface modification, number of attachments per overdenture, type of attachment used, use of a metal structure/cast mesh for reinforcement of the prosthesis, occurrence of implant and/or prosthesis failure, time from implant/prosthesis installation to failure, occurrence of technical complications, follow-up time.

Information on the following technical complications was collected, when available: fracture of acrylic teeth, fracture of prosthesis acrylic base, complete transverse fracture of the overdenture, attachment male or female part loose, attachment female part fracture or worn out, replacement of attachment components, overdenture relining, implant fracture.

Contact with authors for possible missing data was performed.

### Analyses

The mean, standard deviation (SD), and percentage were calculated for the aforementioned variables, whenever suitable. The test performed were the following: Kolmogorov–Smirnov (to evaluate the normal distribution), Levene’s test (to evaluate homoscedasticity), Student’s t-test or Mann-Whitney (for two independent groups, continuous variables), Pearson’s chi-squared or Fisher’s exact test (for categorical variables). The log-rank (Mantel-Cox) test was used to compare the survival distributions of implants between the maxilla and the mandible. The interval survival rate (ISR) of implants and prosthesis was calculated using the information for the period of failure retrieved from the included studies, and the cumulative survival rate (CSR) was calculated over the maximal period of follow-up reported, in a life-table survival analysis. The degree of statistical significance was considered *p* < 0.05. These data were statistically analyzed using the SPSS version 28 software (SPSS Inc., Chicago, IL, USA).

A meta-analysis applying the DerSimonian-Laird random-effects method (DerSimonian and Laird, 1986) was performed to calculate the estimated MBL under different follow-up times. The I^2^ statistic was used to express the percentage of the total variation across studies due to heterogeneity, with 25% corresponding to low heterogeneity, 50% to moderate and 75% to high. A meta-regression assessing the relationship between mean MBL and follow-up was performed. The data were analyzed using the statistical software OpenMeta[Analyst] [35].

## Results

### Literature search

**Fig. 1:**
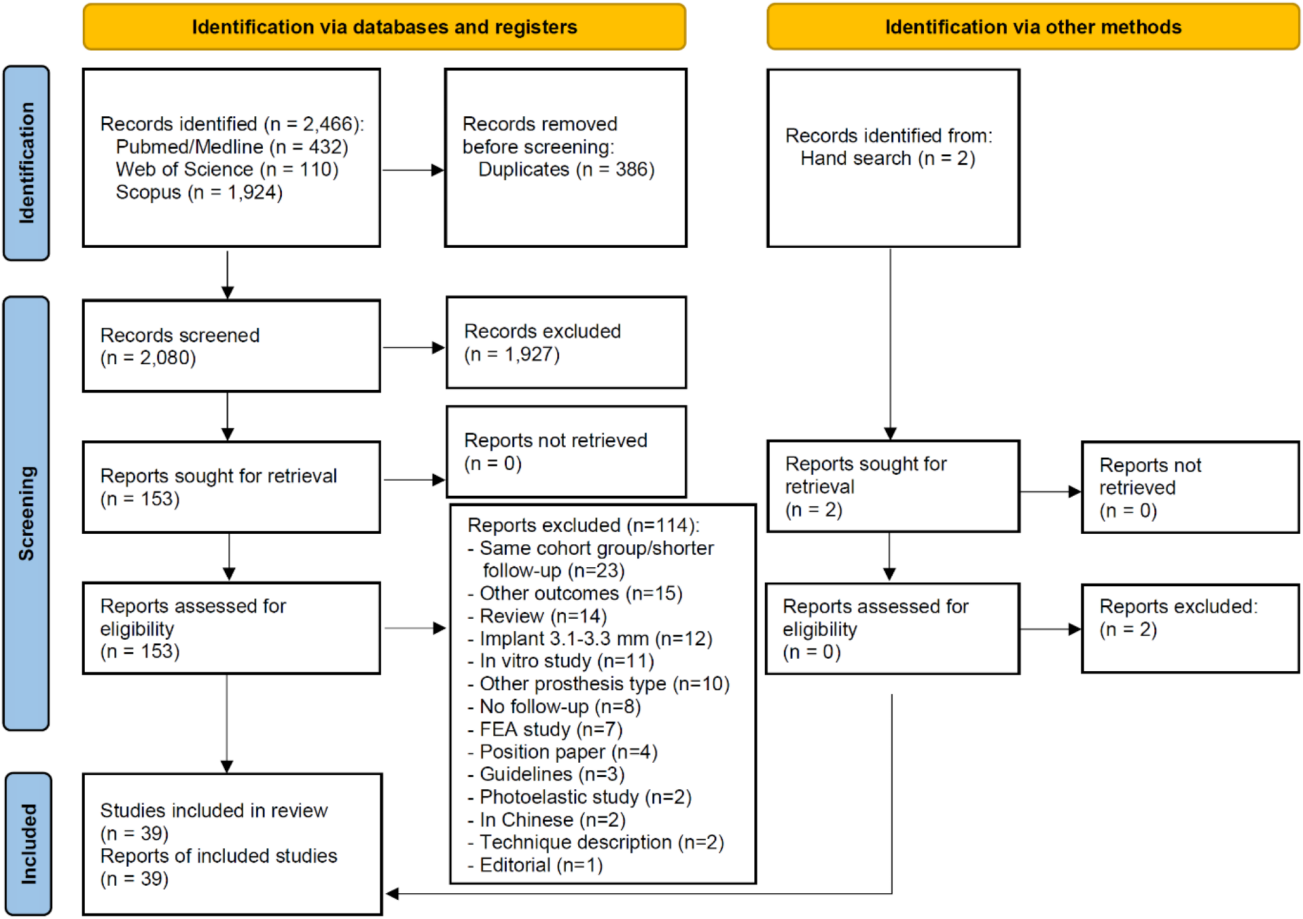
Study screening process

The study selection process is summarized in Fig. 1. The search strategy in the databases resulted in 2,466 papers (432 in PubMed/Medline, 110 in Web of Science, 1,924 in Scopus). A total of 386 articles were cited in more than one database (duplicates). The reviewers independently screened the abstracts for those articles related to the aim of the review, leading to the exclusion of 1,927 articles as the studies were not related to the subject. Of the resulted 153 studies, 114 were excluded due to one or more reasons for exclusion, according to the exclusion criteria. Hand-searching of journals and of the reference lists of selected studies yielded 2 additional papers, not eligible though. Thus, 39 studies were included in the review [10, 18, 19, 36–71].

### Description of the studies

Detailed data on every publication is presented in Table S1, in the Supplementary Material. Table 1 presents the summarized global data of the included studies, also separately between overdentures installed in different jaws.

The 39 included publications reported 3,787 mini-implants supporting 1,026 overdentures (109 in maxillae, 896 in mandibles) in 1,005 patients. Twenty-one patients had mini-implant-retained overdenture in both the maxilla and mandible. The 1,005 patients consisted of 396 men and 496 women, with no information on sex for 114 patients. The mean follow-up was 28.1 ± 19.8 months (min-max 0.3–84.0). A total of 202 mini-implants failed, after a mean of 9.4 ± 11.8 months (min-max 0.3–60.0). Failure at the patient level was 133/1,005 (13.2%). Seventy-six out of 1,026 prostheses were considered as failures, the main reason (*n* = 69; 90.8%) being transversal fracture of the overdenture.

Most of the implants in the mandible were submitted to immediate loading, while in the maxilla the delayed loading protocol was more commonly applied. O-ring-ball was the most commonly used attachment system, followed by Equator, Locator, and bar-clip system. A high rate of transversal fracture of the prosthesis and wear of the attachment parts was observed. Relining of the prosthesis base was a common needed maintenance process.

**Table 1:** Summarized data of the included studies

Variable	Global	Maxilla	Mandible
Implants / Patients (n)	3,787 / 1,005	621 / 109	3,166 / 896
Men / Women, n (%)	396 (44.4) / 495 (55.6)	41 (38.0) / 67 (62.0)	355 (45.3) / 428 (54.7)
Not available	114	1	113
Age (years), mean ± SD (min-max)	66.8 ± 17.1 (6–92)	67.5 ± 15.4 (52–92)	66.4 ± 22.3 (6–92)
Implants per patient, mean ± SD (min-max)	3.8 ± 1.5 (2–12)	6.6 ± 2.0 (2–12)	3.4 ± 1.0 (2–6)
Implants per patient, n patients (%)			
2	271 (27.0)	1 (0.9)	270 (30.1)
3	5 (0.5)	-	5 (0.6)
4	602 (59.9)	11 (10.1)	591 (66.0)
5	35 (3.5)	7 (6.4)	28 (3.1)
6	59 (5.8)	57 (52.3)	2 (0.2)
7	9 (0.9)	9 (8.3)	-
8	9 (0.9)	9 (8.3)	-
10	8 (0.8)	8 (7.3)	-
12	7 (0.7)	7 (6.4)	-
Implants per prosthesis, mean ± SD (min-max)	3.7 ± 1.2 (2–10)	5.7 ± 1.1 (2–10)	3.5 ± 1.0 (2–6)
Implants per prosthesis, n prostheses (%)			
2	257 (26.8)	1 (0.9)	274 (29.9)
3	5 (0.5)	-	3 (0.5)
4	615 (59.9)	19 (17.4)	596 (65.0)
5	48 (4.7)	14 (12.8)	34 (3.7)
6	68 (6.6)	60 (55.1)	8 (0.9)
7	9 (0.9)	9 (8.3)	-
8	5 (0.5)	5 (4.6)	-
10	1 (0.1)	1 (0.9)	-
Follow-up (months), mean ± SD (min-max)	28.1 ± 19.8 (0.3–84.0)	32.9 ± 16.9 (0.8–60.0)	27.1 ± 20.2 (0.3–84.0)
Healing time, n (%)			
Immediate loading	1,961 (64.3)	20 (7.0)	1,941 (70.3)
0.5-3 months	781 (25.6)	76 (26.5)	705 (25.5)
4–6 months	307 (10.1)	191 (66.6)	116 (4.2)
Not available	738	334	404
Implant failure, n/total (%)			
Implant level	202/3,787 (5.3)	69/621 (11.1)	133/3,166 (4.2)
Patient level	133/1,005 (13.2)	37/109 (33.9)	96/896 (10.7)
Time of failure (months), mean ± SD (min-max)	9.4 ± 11.8 (0.3–60.0)	10.0 ± 10.7 (0.8–39.0)	9.1 ± 12.3 (0.3–60.0)
Mandibular			
Primary failure, n (%) ^a^			
Immediate loading	-/93	-/13	-/80
Healing 0.5-3 months	20/69 (29.0)	1/16 (6.3)	19/53 (35.8)
Healing 4–6 months	32/40 (80.0)	32/40 (80.0)	-
Failed implant replaced by a new one ^b^, n/total (%)	47/80 (58.8)	20/46 (43.5)	27/34 (79.4)
Prosthesis failure, n/total (%)	76/1,026 (7.4)	16/109 (14.7)	60/917 (6.5)
Time of failure ^c^ (months), mean ± SD (min-max)	21.8 ± 17.8 (2–60)	16.5 ± 10.8 (2–27)	23.3 ± 19.3 (3–60)
Reason for prosthesis failure, n (%)			
Prosthesis fracture	69 (90.8)	12 (75.0)	57 (95.0)
Loss of implants	6 (7.9)	3 (18.8)	3 (5.0)
Received additional implants in order to make a fixed full-arch prosthesis	1 (1.3)	1 (6.2)	-
Attachment system, n prostheses (%)			
O’Ring-Ball	776 (75.6)	103 (94.5)	673 (73.4)
Equator	114 (11.1)	4 (3.7)	110 (12.0)
Locator	113 (11.0)	2 (1.8)	111 (12.1)
Bar-clip	23 (2.3)	-	23 (2.5)
Technical complications, n/total (%) ^b^			
Fracture of acrylic teeth	16/276 (5.8)	3/56 (5.4)	13/220 (5.9)
Fracture of acrylic base	12/255 (4.7)	0/56 (0)	12/199 (6.0)
Transversal fracture of prosthesis	69/477 (14.5)	12/69 (17.4)	57/408 (14.0)
Attachment– matrix part			
Fracture	9/1,433 (0.6)	0/340 (0)	9/1,093 (0.8)
Loose	6/1,269 (0.5)	0/340 (0)	6/929 (0.6)
Fell off	33/1,786 (1.8)	9/365 (2.5)	24/1,421 (1.7)
Wear	250/1,301 (19.2)	0/340 (0)	250/961 (26.0)
Attachment– patrix part			
Fracture	1/1,353 (0.1)	0/340 (0)	1/1,013 (0.1)
Wear	49/250 (19.6)	0/6 (0)	49/244 (20.1)
New component	186/1,687 (11.0)	9/340 (2.6)	177/1,347 (13.1)
Relining	105/437 (24.0)	14/69 (20.3)	91/368 (24.7)
New overdenture	23/79 (29.1)	-	23/79 (29.1)

^a^ Failure up till prosthetic loading

^b^ For the cases with available information

^c^ Time of prosthesis failure was available for only 19 cases

### Analyses

A total of 202 mini-implants were considered as failure, with a clear concentration of failures in the first year of follow-up (Table 2). The 7-year CSR was 91.4%. The CSR was lower at 5 years for mini-implants placed in the maxilla (87.1%, Table 3) in comparison to mini-implants placed in the mandible (92.3%, Table 4), and the difference in survival was statistically significant (*p* < 0.001; Log-rank test).

The survival of implants submitted to early/delayed loading was lower than implants immediately loaded (*p* < 0.001; Log-rank test), which was true even when only mandibular implants were considered (*p* = 0.005; Log-rank test). This, however, was the opposite when only maxillary implants were considered (*p* < 0.001; Log-rank test).

When only mandibular overdentures were considered, there was a higher implant failure rate (*p* < 0.001; Log-rank test) among 2-mini-implant retained overdenture (43/548, 92.2% survival) in comparison to 4-mini-implant retained overdenture (90/2384, 96.2% survival).

Unfortunately, information about the time point of failure for the overdentures was available for only 19 of the 76 failures. Therefore, a life-table analysis for the prostheses was not carried out.

**Table 2:** Life-table survival analysis showing the cumulative survival rate of mini-implants to support overdentures

Interval start time (years)	Number entering interval	Number withdrawing during interval	Number exposed to risk	Implant failures	Survival rate within each interval– ISR (%)	Cumulative proportion surviving at end of interval– CSR (%)	SE
0	3787	322	3626.0	114	96.9	96.9	0.3
1	3351	1124	2789.0	54	98.1	95.0	0.4
2	2173	1094	1626.0	14	99.1	94.2	0.4
3	1065	277	926.5	14	98.5	92.7	0.6
4	774	120	714.0	1	99.9	92.6	0.6
5	653	548	379.0	5	98.7	91.4	0.8
6	100	86	57.0	0	100.0	91.4	0.8
7	14	14	7.0	0	100.0	91.4	0.8

ISR - interval survival rate, CSR - cumulative survival rate, SE– standard error

**Table 3:** Life-table survival analysis showing the cumulative survival rate of mini-implants to support overdentures in the maxilla

Interval start time (years)	Number entering interval	Number withdrawing during interval	Number exposed to risk	Implant failures	Survival rate within each interval– ISR (%)	Cumulative proportion surviving at end of interval– CSR (%)	SE
0	621	6	618.0	45	92.7	92.7	1.0
1	570	30	555.0	14	97.5	90.4	1.2
2	526	319	366.5	7	98.1	88.7	1.3
3	200	52	174.0	3	98.3	87.1	1.6
4	145	0	145.0	0	100.0	87.1	1.6
5	145	145	72.5	0	100.0	87.1	1.6

ISR - interval survival rate, CSR - cumulative survival rate, SE– standard error

**Table 4:** Life-table survival analysis showing the cumulative survival rate of mini-implants to support overdentures in the mandible

Interval start time (years)	Number entering interval	Number withdrawing during interval	Number exposed to risk	Implant failures	Survival rate within each interval– ISR (%)	Cumulative proportion surviving at end of interval– CSR (%)	SE
0	3166	316	3008.0	69	97.7	97.7	0.3
1	2781	1094	2234.0	40	98.2	96.0	0.4
2	1647	775	1259.5	7	99.4	95.4	0.4
3	865	225	752.5	11	98.5	94.0	0.6
4	629	120	569.0	1	99.8	93.9	0.6
5	508	403	306.5	5	98.4	92.3	0.9
6	100	86	57.0	0	100.0	92.3	0.9
7	14	14	7.0	0	100.0	92.3	0.9

ISR - interval survival rate, CSR - cumulative survival rate, SE– standard error

Table 5 shows the results of the meta-analyses for the outcome MBL under different follow-ups. The estimated mean MBL gradually increased from 0.518 mm (≤ 6 months) to 1.260 mm (58.8–90 months).

**Table 5:** DerSimonian-Laird random-effects model analysis for MBL under different follow-ups

Follow-up (months)	Studies*/ implants (*n*)	MBL estimate (95% CI) (mm)	SE	*p* value	Heterogeneity
≤ 6	8/748	0.518 (0.382, 0.654)	0.069	< 0.001	τ^2^ = 0.037, *p* < 0.001, I^2^ = 96.914
12	14/951	0.655 (0.405, 0.905)	0.127	< 0.001	τ^2^ = 0.217, *p* < 0.001, I^2^ = 98.557
18–24	7/330	0.878 (0.460, 1.296)	0.213	< 0.001	τ^2^ = 0.306, *p* < 0.001, I^2^ = 97.966
36	5/360	0.948 (0.494, 1.402)	0.232	< 0.001	τ^2^ = 0.263, *p* < 0.001, I^2^ = 98.460
58.9–80	6/408	1.260 (0.940, 1.580)	0.163	< 0.001	τ^2^ = 0.132, *p* < 0.001, I^2^ = 93.239

MBL– marginal bone loss, 95% CI– 95% confidence interval, SE– standard error

* When data on MBL for all implants in a study was not available (as a global mean value), then data on the mean value of the different sub-groups of study was entered. In these cases, each sub-group was considered as one “study”

**Fig. 2:**
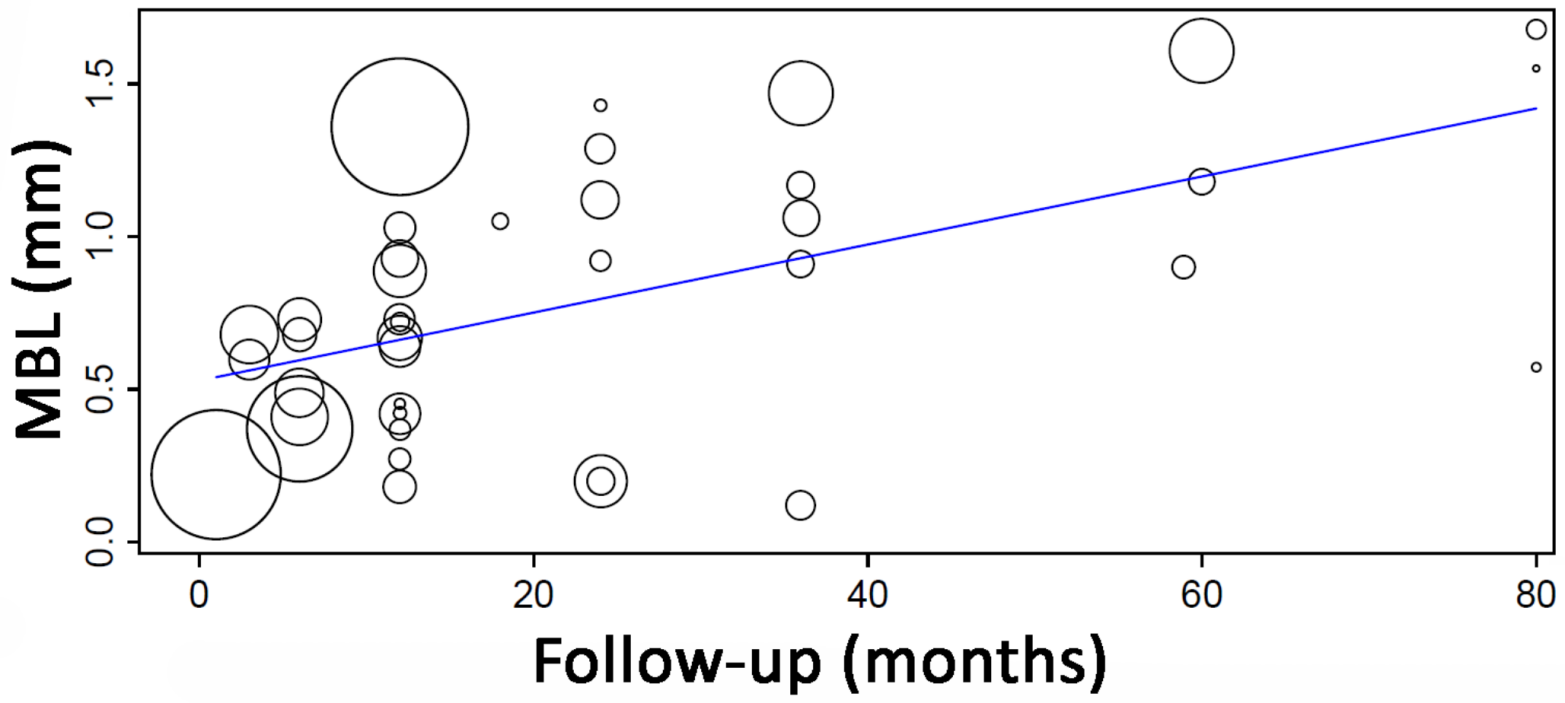
Scatter plot for the meta-regression with the association between follow-up (in months) and mean marginal bone loss (MBL). Positive values mean bone loss, while the negative values mean bone gain. Every circle represents a study or a different follow-up point in a same study, and the size of the circle represents the weight of the study in the analysis. The blue line represents the fitted line plot

A meta-regression considering the effect of follow-up on the mean MBL (Fig. 2) resulted in the following first-degree equation:

y = 0.528 + 0.011x, where:

Intercept = 0.528 (0.362, 0.694), standard error 0.085, *p* < 0.001.

Follow-up = 0.011 (0.006, 0.017), standard error 0.003, *p* < 0.001.

There was an estimated increase of 0.011 mm in MBL for every additional month of follow-up, with statistical significance.

### Quality assessment

All included studies were classified as “good” according to the quality assessment tool (see Tables S2 and S3, Supplementary Material). In most cases the main issues in the publications were related to not well-described statistical methods, and to the inclusion of non-consecutive patients in the studies. These issues were not a problem though, since there was information about follow-up as well as the main outcome, namely, mini-implant failure.

## Discussion

The purpose of the present review was to assess the clinical outcomes of overdentures retained by dental mini-implants. The results showed that mini-implants used to retain/support overdentures present a high CSR over 7 years, namely 91.4%.

Most of the implant failures happened during the first year. Moreover, a high rate of primary failure (up to prosthetic loading) was observed among the implants that were not submitted to immediate loading. A considerable percentage of implants fail in the early period after implant installation [72], regardless of the follow-up time [73]. One has to consider that a reduced implant diameter means a reduction in the bone-implant contact surface area, which could compromise the osseointegration process [74], or not be enough to withstand loading forces [75]. Furthermore, mini-implants usually are the option for rehabilitation in sites with poorer bone quality and lack of bone volume, which may not only directly influence the implant initial stability values [76], but also statistically affect implant survival rates in a negative manner [77]. All this may have a substantial impact on prosthetic rehabilitation plan [78], since the rehabilitation with mini-implants may be already seen as the last resource for an implant-retained prosthesis, due to the scarcity of available bone, without the need of more invasive approaches such as bone grafting procedures.

Immediate loading of the mini-implants was common for overdentures in the mandible, but very uncommon for overdentures in the maxilla. This may be related to the maxillary bone being more trabecular and softer than the mandibular bone, which is denser (Lekholm and Zarb, 1985), which in turn is generally perceived as resulting in lower primary stability, greater micromotion, and consequently a greater risk of maxillary implants to osseointegrate when submitted to immediate prosthetic loading [77, 79–81]. In fact, it was observed in the present results that the survival of immediately loaded implants was lower than implants submitted to early/delayed loading, when only the maxilla was considered. Due to the reduced diameter of mini-implants, it may be prudent to undersize the preparation of the implant bone sites, as well as to do not immediately load the implants when the clinically perceived primary stability is lower than an insertion torque value of 10 Ncm and an resonance frequency analysis of 60 [82]. According to a recent review, however, implants supporting fixed prostheses (not overdentures) in the maxilla subjected to immediate loading present high survival rates [83].

When only mandibular overdentures were considered, there was lower implant survival for 2-mini-implant-retained in comparison to 4-mini-implant-retained mandibular overdenture. It has been suggested that 2-mini-implant-retained mandibular overdenture could be used for cost-effectiveness clinical situations [41], although a 4-mini-implant-retained overdenture is associated with higher patient satisfaction [43]. Moreover, a FEA study observed that an overdenture retained by 4 implants is steadier than one retained by 2 implants when functioning with the anterior teeth. In the same study it was also observed that under the simulated action of cutting food with the anterior teeth, the maximum stress value in the abutments with the 2-implant model was three-fold than with the 4-implant model, suggesting that higher risk of damage to the abutments in 2-implant overdentures [84]. Due to the aforementioned reasons, the use of 4 mini-implants to retain mandibular overdentures is recommended instead of only 2 mini-implants.

The attachment system most commonly used was of the O-ring-ball system. This can be associated with narrow diameter of the mini-implants, which limits the space for wider, more elaborated, attachment systems. And also, to the fact that mini-implants are usually commercialized as one-piece implants.

There was a high rate of transversal fracture of the prosthesis, which usually happens due to the thinning and weakening of the acrylic resin bases in the area of the prosthesis that harbors the matrix part of the attachment systems [85]. This type of fracture is commonly reported by studies in which existing complete dentures are transformed into implant-retained overdentures [86]. Metal frameworks could minimize the occurrence of overdenture fractures [57].

Considering the relatively high rate of some technical complications, it can be said that mini-implant-retained overdentures may require frequent maintenance follow-ups to address the complications, which is usual with implant-retained overdentures [87, 88]. It has been suggested that substituting the direct chairside attachment matrix pick-up technique with an indirect laboratory procedure may reduce the number of complication events and consequently of maintenance appointments [89].

A high rate of prosthesis relining was observed, something that is expected in cases of immediate loading (the majority of the cases observed in the review), probably due to progressive subsiding of the postoperative swelling [88]. This must be weighed with the suggestion that immediate loading may result in lower postoperative symptoms and lower levels of pain and functional discomfort during the 6-week healing period [16].

Regarding MBL, a finite element analysis (FEA) study showed that the stresses in cortical bone decrease in inverse proportion to the increase in implant diameter with both vertical and lateral load [90]. More specific to mini-implants, stress and strain occurring are mainly localized at the cortical bone around the bone-implant interface [91], with higher bone loading in comparison to standard diameter implants, which could increase the risk of MBL around mini-implants [92]. However, the present results seem to indicate that MBL around mini-implants retaining overdentures present acceptable results in relation to wider diameter implants [93].

Limitations of the present review include the fact that there was a considerable number of confounding factors. There was no information about how many implants were inserted and failed in several different conditions for most (if not all) of the studies. Studies reported the presence of diabetics among the patients, as well as smokers, bruxers, and patients taking selective serotonin reuptake inhibitors, proton-pump inhibitors, or bisphosphonates, which are medications commonly prescribed for the elderly. All these factors could have had a considerable impact on implant failure rates [94–99]. Furthermore, the retrospective nature of many studies results in flaws manifested by the gaps in information. In addition, several studies presented small cohort sizes and short follow-ups. The global number of mini-implant supported overdentures installed in the maxilla reported in the literature is still small, so more studies are needed in order to draw more robust conclusions.

Based on present results, it can be recommended the use of 4 mini-implants in the mandible for retaining overdenture prostheses, as there is a better implant survival prognosis for this option in comparison to when only 2 mini-implants are used. Mini-implants should not be immediately loaded when the clinically perceived primary stability is low, something that can be quantitatively verified by the values of insertion torque and resonance frequency analysis. Due to the high rate of prosthesis transversal fracture, it is recommended the manufacture of overdentures with metal frameworks, which could minimize the occurrence of this complication. It is recommended that dentists maintain a regular and close follow-up of patients rehabilitated with this mini-implant supported overdentures, due to the expected high prevalence of technical complications. Moreover, substituting the direct chairside attachment matrix pick-up technique with an indirect laboratory procedure may reduce the number of complication events and consequently of maintenance appointments.

## Conclusions

Although presenting a high 7-year CSR and acceptable MBL, mini-implant-retained overdentures may require frequent maintenance follow-ups, due to the high rate of technical complications.

## Electronic supplementary material

Below is the link to the electronic supplementary material.

Supplementary Material 1

## Data Availability

No datasets were generated or analysed during the current study.

## References

[1] UN (2022) United Nations. World Population prospects 2022: Summary of results. Book title, 27th edn. United Nations Department of Economic and Social Affairs, Population Division

[2] Dye BA, Weatherspoon DJ, Lopez Mitnik G (2019) Tooth loss among older adults according to poverty status in the United States from 1999 through 2004 and 2009 through 2014. Journal of the American Dental Association (1939) 150:9–23.e3. 10.1016/j.adaj.2018.09.010 10.1016/j.adaj.2018.09.010 PMC6394416 30503018

[3] Petersen PE, Kandelman D, Arpin S, Ogawa H (2010) Global oral health of older people–call for public health action. Community Dent Health 27:257–267 21313969

[4] Borg-Bartolo R, Roccuzzo A, Molinero-Mourelle P, Schimmel M, Gambetta-Tessini K, Chaurasia A, Koca-Ünsal RB, Tennert C, Giacaman R, Campus G (2022) Global prevalence of edentulism and dental caries in middle-aged and elderly persons: a systematic review and meta-analysis. J Dent 127:104335. 10.1016/j.jdent.2022.104335 36265526 10.1016/j.jdent.2022.104335

[5] Boyne PJ (1966) Osseous repair of the postextraction alveolus in man. Oral surgery, oral medicine, and oral pathology 21:805– 13. 10.1016/0030-4220(66)90104-6 10.1016/0030-4220(66)90104-6 5219671

[6] Devlin H, Sloan P (2002) Early bone healing events in the human extraction socket. Int J Oral Maxillofac Surg 31:641–645. 10.1054/ijom.2002.0292 12521322 10.1054/ijom.2002.0292

[7] Carlsson GE, Persson G (1967) Morphologic changes of the mandible after extraction and wearing of dentures. A longitudinal, clinical, and x-ray cephalometric study covering 5 years. Odontologisk revy 18:27–54 5227389

[8] Chrcanovic BR, Abreu MH, Custodio AL (2011) Morphological variation in dentate and edentulous human mandibles. Surg Radiol Anat 33:203–213. 10.1007/s00276-010-0731-4 20878404 10.1007/s00276-010-0731-4

[9] LaBarre E, Giusti L, Pitigoi-Aron G (2007) Addressing problems in complete dentures. Compendium of continuing education in dentistry (Jamesburg, NJ: 1995) 28:538– 40, 542 18018388

[10] Catalán A, Martínez A, Marchesani F, González U (2016) Mandibular overdentures retained by two mini-implants: a Seven-Year Retention and satisfaction study. J Prosthodontics: Official J Am Coll Prosthodontists 25:364–370. 10.1111/jopr.12373 10.1111/jopr.12373 26422523

[11] Budtz-Jørgensen E (1981) Oral mucosal lesions associated with the wearing of removable dentures. J Oral Pathol 10:65–80. 10.1111/j.1600-0714.1981.tb01251.x 6792333 10.1111/j.1600-0714.1981.tb01251.x

[12] Raghoebar GM, Batenburg RH, Vissink A (1999) [Local bone augmentations for the use of implants]. Nederlands Tijdschrift voor Tandheelkunde 106:191–194 11930366

[13] Rissolo AR, Bennett J (1998) Bone grafting and its essential role in implant dentistry. Dental Clin N Am 42:91–116 9421672

[14] Sanz-Sánchez I, Sanz-Martín I, Ortiz-Vigón A, Molina A, Sanz M (2022) Complications in bone-grafting procedures: classification and management. Periodontol 2000 88:86–102. 10.1111/prd.12413 35103322 10.1111/prd.12413

[15] (2017) The Glossary of Prosthodontic terms: Ninth Edition. J Prosthet Dent 117:e1–e105. 10.1016/j.prosdent.2016.12.001 10.1016/j.prosdent.2016.12.001 28418832

[16] Leles CR, de Paula MS, Curado TFF, Silva JR, Leles JLR, McKenna G, Schimmel M (2022) Flapped versus flapless surgery and delayed versus immediate loading for a four mini implant mandibular overdenture: a RCT on post-surgical symptoms and short-term clinical outcomes. Clin Oral Implants Res 33:953–964. 10.1111/clr.13974 35818640 10.1111/clr.13974

[17] Upendran A, Gupta N, Salisbury HG (2023) Dental Mini-Implants. Book title. StatPearls Publishing Copyright © 2023, StatPearls Publishing LLC., Treasure Island (FL) ineligible companies. Disclosure: Neha Gupta declares no relevant financial relationships with ineligible companies. Disclosure: Herb Salisbury declares no relevant financial relationships with ineligible companies 30020638

[18] Griffitts TM, Collins CP, Collins PC (2005) Mini dental implants: an adjunct for retention, stability, and comfort for the edentulous patient. Oral Surg Oral Med Oral Pathol Oral Radiol Endod 100(e81–4). 10.1016/j.tripleo.2005.06.018 10.1016/j.tripleo.2005.06.018 16243233

[19] Tomasi C, Idmyr BO, Wennström JL (2013) Patient satisfaction with mini-implant stabilised full dentures. A 1-year prospective study. J Rehabil 40:526–534. 10.1111/joor.12053 10.1111/joor.12053 23551029

[20] Chrcanovic BR, Kisch J, Albrektsson T, Wennerberg A (2018) Factors influencing the fracture of dental implants. Clin Implant Dent Relat Res 20:58–67. 10.1111/cid.12572 29210188 10.1111/cid.12572

[21] Meijer HJ, Starmans FJ, Steen WH, Bosman F (1996) Loading conditions of endosseous implants in an edentulous human mandible: a three-dimensional, finite-element study. J Rehabil 23:757–763. 10.1046/j.1365-2842.1996.d01-185.x 10.1046/j.1365-2842.1996.d01-185.x 8953480

[22] Telles LH, Portella FF, Rivaldo EG (2019) Longevity and marginal bone loss of narrow-diameter implants supporting single crowns: a systematic review. PLoS ONE 14:e0225046. 10.1371/journal.pone.0225046 31710656 10.1371/journal.pone.0225046 PMC6844460

[23] Gaszynska E, Szatko F, Godala M, Gaszynski T (2014) Oral health status, dental treatment needs, and barriers to dental care of elderly care home residents in Lodz, Poland. Clin Interv Aging 9:1637–1644. 10.2147/cia.s69790 25284997 10.2147/CIA.S69790 PMC4181440

[24] Renvert S, Quirynen M (2015) Risk indicators for peri-implantitis. A narrative review. Clin oral Implants Res 26 Suppl 11:15–44. 10.1111/clr.12636 10.1111/clr.12636 26385619

[25] Vi S, Pham D, Du YYM, Arora H, Tadakamadla SK (2021) Mini-Implant-Retained Overdentures for the Rehabilitation of Completely Edentulous Maxillae: A Systematic Review and Meta-Analysis. International journal of environmental research and public health 18. 10.3390/ijerph18084377 10.3390/ijerph18084377 PMC8074399 33924167

[26] Jawad S, Clarke PT (2019) Survival of Mini Dental implants used to Retain Mandibular Complete overdentures: systematic review. Int J Oral Maxillofac Implants 34:343–356. 10.11607/jomi.6991 30883617 10.11607/jomi.6991

[27] Page MJ, Moher D, Bossuyt PM, Boutron I, Hoffmann TC, Mulrow CD, Shamseer L, Tetzlaff JM, Akl EA, Brennan SE, Chou R, Glanville J, Grimshaw JM, Hróbjartsson A, Lalu MM, Li T, Loder EW, Mayo-Wilson E, McDonald S, McGuinness LA, Stewart LA, Thomas J, Tricco AC, Welch VA, Whiting P, McKenzie JE (2021) PRISMA 2020 explanation and elaboration: updated guidance and exemplars for reporting systematic reviews. BMJ (Clinical Res ed) 372:n160. 10.1136/bmj.n160 10.1136/bmj.n160 PMC8005925 33781993

[28] Chrcanovic BR, Albrektsson T, Wennerberg A (2016) Dental implants in irradiated versus nonirradiated patients: a meta-analysis. Head Neck 38:448–481. 10.1002/hed.23875 25242560 10.1002/hed.23875

[29] Chrcanovic BR, Reher P, Sousa AA, Harris M (2010) Osteoradionecrosis of the jaws–a current overview–part 1: physiopathology and risk and predisposing factors. Oral Maxillofacial Surg 14:3–16. 10.1007/s10006-009-0198-9 10.1007/s10006-009-0198-9 20119841

[30] Chrcanovic BR, Reher P, Sousa AA, Harris M (2010) Osteoradionecrosis of the jaws–a current overview–part 2: dental management and therapeutic options for treatment. Oral Maxillofacial Surg 14:81–95. 10.1007/s10006-010-0205-1 10.1007/s10006-010-0205-1 20145963

[31] NIH (2014) Quality Assessment Tool for Case Series studies. National Institutes of Health (NIH). Accessed Acces Date. https://www.nhlbi.nih.gov/health-topics/study-quality-assessment-tools

[32] Riley DS, Barber MS, Kienle GS, Aronson JK, von Schoen-Angerer T, Tugwell P, Kiene H, Helfand M, Altman DG, Sox H, Werthmann PG, Moher D, Rison RA, Shamseer L, Koch CA, Sun GH, Hanaway P, Sudak NL, Kaszkin-Bettag M, Carpenter JE, Gagnier JJ (2017) CARE guidelines for case reports: explanation and elaboration document. J Clin Epidemiol 89:218–235. 10.1016/j.jclinepi.2017.04.026 28529185 10.1016/j.jclinepi.2017.04.026

[33] Tonetti MS, Schmid J (1994) Pathogenesis of implant failures. Periodontol 2000 4:127–138. 10.1111/j.1600-0757.1994.tb00013.x 10.1111/j.1600-0757.1994.tb00013.x 9673201

[34] Albrektsson T, Chrcanovic B, Östman PO, Sennerby L (2017) Initial and long-term crestal bone responses to modern dental implants. Periodontol 2000 73:41–50. 10.1111/prd.12176 28000272 10.1111/prd.12176

[35] Wallace BC, Dahabreh IJ, Trikalinos TA, Lau J, Trow P, Schmid CH (2012) Closing the gap between methodologists and End-Users: R as a computational back-end. J Stat Softw 49:1–15

[36] Ahn MR, An KM, Choi JH, Sohn DS (2004) Immediate loading with mini dental implants in the fully edentulous mandible. Implant Dent 13:367–372. 10.1097/01.id.0000148560.65514.3d 15591999 10.1097/01.id.0000148560.65514.3d

[37] Araujo CR, Martins-Junior PA, Araujo RC, Sa MA, Wassall T, Ferreira AJ (2015) Narrow-implant-retained overdenture in an atrophic mandibular ridge: a case report with 6-year follow-up. Gen Dent 63:e12–e15 26545281

[38] Bellia E, Boggione L, Terzini M, Manzella C, Menicucci G (2018) Immediate Loading of Mandibular overdentures retained by two mini-implants: a Case Series Preliminary Report. Int J Prosthodont 31:558–564. 10.11607/ijp.5589 30339161 10.11607/ijp.5589

[39] Bielemann AM, Schuster AJ, Possebon A, Schinestsck AR, Chagas-Junior OL, Faot F (2022) Clinical performance of narrow-diameter implants with hydrophobic and hydrophilic surfaces with mandibular implant overdentures: 1-year results of a randomized clinical trial. Clin Oral Implants Res 33:21–32. 10.1111/clr.13851 34551146 10.1111/clr.13851

[40] Brandt R, Hollis S, Ahuja S, Adatrow P, Balanoff W (2012) Short-term objective and subjective evaluation of small-diameter implants used to support and retain mandibular prosthesis. J Tenn Dent Assoc 92:34– 8; quiz 38– 9. 22870551

[41] Chatrattanarak W, Aunmeungtong W, Khongkhunthian P (2022) Comparative clinical study of conventional dental implant and mini dental implant-retained mandibular overdenture: a 5- to 8-Year prospective clinical outcomes in a previous randomized clinical trial. Clin Implant Dent Relat Res 24:475–487. 10.1111/cid.13098 35675561 10.1111/cid.13098

[42] Curado TFF, Silva JR, Nascimento LN, Leles JLR, McKenna G, Schimmel M, Leles CR (2023) Implant survival/success and peri-implant outcomes of titanium-zirconium mini implants for mandibular overdentures: results from a 1-year randomized clinical trial. Clin Oral Implants Res 34:769–782. 10.1111/clr.14102 37254798 10.1111/clr.14102

[43] de Souza RF, Ribeiro AB, Della Vecchia MP, Costa L, Cunha TR, Reis AC, Albuquerque RF Jr (2015) Mini vs. Standard Implants for Mandibular Overdentures: a Randomized Trial. J Dent Res 94:1376–1384. 10.1177/0022034515601959 26294416 10.1177/0022034515601959

[44] Elsyad MA (2016) Patient satisfaction and prosthetic aspects with mini-implants retained mandibular overdentures. A 5-year prospective study. Clin Oral Implants Res 27:926–933. 10.1111/clr.12660 26129836 10.1111/clr.12660

[45] Enkling N, Moazzin R, Geers G, Kokoschka S, Abou-Ayash S, Schimmel M (2020) Clinical outcomes and bone-level alterations around one-piece mini dental implants retaining mandibular overdentures: 5-year follow-up of a prospective cohort study. Clin Oral Implants Res 31:549–556. 10.1111/clr.13591 32096255 10.1111/clr.13591

[46] Hussein MO, Alruthea MS (2020) Marginal bone level changes and oral Health Impact Profile (14) score of smokers treated by Mandibular Mini Implant overdentures: a 5-Year follow-up study. Eur J Dentistry 14:590–597. 10.1055/s-0040-1714763 10.1055/s-0040-1714763 PMC7535974 32777837

[47] Jawad S, Barclay C, Whittaker W, Tickle M, Walsh T (2017) A pilot randomised controlled trial evaluating mini and conventional implant retained dentures on the function and quality of life of patients with an edentulous mandible. BMC Oral Health 17:53. 10.1186/s12903-017-0333-1 28202072 10.1186/s12903-017-0333-1 PMC5310054

[48] Jofré J, Conrady Y, Carrasco C (2010) Survival of splinted mini-implants after contamination with stainless steel. Int J Oral Maxillofac Implants 25:351–356 20369095

[49] Kabbua P, Aunmeungtong W, Khongkhunthian P (2020) Computerised occlusal analysis of mini-dental implant-retained mandibular overdentures: a 1-year prospective clinical study. J Rehabil 47:757–765. 10.1111/joor.12969 10.1111/joor.12969 32242956

[50] Kilic S, Altintas SH, Yilmaz Altintas N, Ozkaynak O, Bayram M, Kusgoz A, Taskesen F (2017) Six-year survival of a Mini Dental Implant-retained overdenture in a child with ectodermal dysplasia. J Prosthodontics: Official J Am Coll Prosthodontists 26:70–74. 10.1111/jopr.12366 10.1111/jopr.12366 26418841

[51] Kovacic I, Persic S, Kranjcic J, Lesic N, Celebic A (2018) Rehabilitation of an extremely resorbed Edentulous Mandible by short and narrow Dental implants. Case reports in dentistry 2018:7597851. 10.1155/2018/7597851 10.1155/2018/7597851 PMC6317120 30671267

[52] Kumari P, Verma M, Sainia V, Gupta A, Gupta R, Gill S (2016) Mini-implants, mega solutions: a Case Series. J Prosthodontics: Official J Am Coll Prosthodontists 25:682–686. 10.1111/jopr.12382 10.1111/jopr.12382 26618277

[53] Kämmerer PW, Wolf JM, Buttchereit I, Frerich B, Ottl P (2021) Prospective clinical implementation of optional implant treatment into pregraduate dental education-mini implants for retention and support of mandibular overdentures. Int J Implant Dentistry 7:87. 10.1186/s40729-021-00371-6 10.1186/s40729-021-00371-6 PMC8429539 34505196

[54] MA EL, Abdraboh AE, Aboelnagga MM, Ghali RM, Lebshtien IT (2019) Effect of low-level laser irradiation on Stability and marginal bone of narrow implants retaining overdentures in moderately controlled Diabetic patients. J Oral Implantol 45:391–397. 10.1563/aaid-joi-D-18-00263 31389750 10.1563/aaid-joi-D-18-00263

[55] Maryod WH, Ali SM, Shawky AF (2014) Immediate versus early loading of mini-implants supporting mandibular overdentures: a preliminary 3-year clinical outcome report. Int J Prosthodont 27:553–560. 10.11607/ijp.3845 25390870 10.11607/ijp.3845

[56] Mifsud DP, Cortes ARG, Zarb MJ, Attard NJ (2020) Maintenance and risk factors for fractures of overdentures using immediately loaded conventional diameter or mini implants with Locator abutments: a cohort study. Clin Implant Dent Relat Res 22:706–712. 10.1111/cid.12952 33094529 10.1111/cid.12952

[57] Mundt T, Schwahn C, Stark T, Biffar R (2015) Clinical response of edentulous people treated with mini dental implants in nine dental practices. Gerodontology 32:179–187. 10.1111/ger.12066 23859086 10.1111/ger.12066

[58] Park JH, Shin SW, Lee JY (2023) Mini-implant mandibular overdentures under a two-step immediate loading protocol: a 4-6-year retrospective study. Gerodontology 40:501–508. 10.1111/ger.12683 37061876 10.1111/ger.12683

[59] Possebon A, Schuster AJ, Chagas-Júnior OL, Pinto LR, Faot F (2021) Prosthetic aftercare, mastication, and quality of life in mandibular overdenture wearers with narrow implants: a 3-year cohort study. J Dent 115:103880. 10.1016/j.jdent.2021.103880 34740638 10.1016/j.jdent.2021.103880

[60] Preoteasa E, Imre M, Preoteasa CT (2014) A 3-year follow-up study of overdentures retained by mini-dental implants. Int J Oral Maxillofac Implants 29:1170–1176. 10.11607/jomi.3222 25216145 10.11607/jomi.3222

[61] Rujiraphan T, Suphangul S, Amornsettachai P, Thiradilok S, Panyayong W (2021) Clinical outcomes of small-diameter implant-retained overdentures: a retrospective analysis. J Osseointegr 13:191–197

[62] Scarano A, Murmura G, Carinci F, Lauritano D (2012) Immediately loaded small-diameter dental implants: evaluation of retention, stability and comfort for the edentulous patient. Eur J Inflamm 10:19–23

[63] Scepanovic M, Calvo-Guirado JL, Markovic A, Delgardo-Ruiz R, Todorovic A, Milicic B, Misic T (2012) A 1-year prospective cohort study on mandibular overdentures retained by mini dental implants. Eur J Oral Implantol 5:367–379 23304690

[64] Schwindling FS, Schwindling FP (2016) Mini dental implants retaining mandibular overdentures: a dental practice-based retrospective analysis. J Prosthodontic Res 60:193–198. 10.1016/j.jpor.2015.12.005 10.1016/j.jpor.2015.12.005 26783089

[65] Temizel S, Heinemann F, Dirk C, Bourauel C, Hasan I (2017) Clinical and radiological investigations of mandibular overdentures supported by conventional or mini-dental implants: a 2-year prospective follow-up study. J Prosthet Dent 117:239–246e2. 10.1016/j.prosdent.2016.07.022 27671375 10.1016/j.prosdent.2016.07.022

[66] Topic J, Poljak-Guberina R, Persic-Kirsic S, Kovacic I, Petricevic N, Popovac A, Celebic A (2022) Adaptation to New dentures and 5 years of clinical use: a comparison between complete denture and mini-implant mandibular overdenture patients based on oral health-related quality of life (OHRQoL) and Orofacial Esthetics. Acta Stomatol Croatica 56:132–142. 10.15644/asc56/2/4 10.15644/asc56/2/4 PMC9262111 35821720

[67] Van Doorne L, Vandeweghe S, Matthys C, Vermeersch H, Bronkhorst E, Meijer G, De Bruyn H (2023) Five years clinical outcome of maxillary mini dental implant overdenture treatment: a prospective multicenter clinical cohort study. Clin Implant Dent Relat Res 25:829–839. 10.1111/cid.13233 37309711 10.1111/cid.13233

[68] Worni A, Fehmer V, Zimmermann P, Sailer I (2020) [Immediate loading of ø 2,4 mm narrow-diameter implants in the edentulous maxilla and mandible]. Swiss Dent J 130:691–698 32909726 10.61872/sdj-2020-09-03

[69] Yilmaz B, Schimmel M, Zimmermann P, Janner S (2020) Use of a New-Generation Mini-implant and attachment system for fabrication of a Maxillary overdenture. Int J Prosthodont 33:576–581. 10.11607/ijp.6544 32956440 10.11607/ijp.6544

[70] Zygogiannis K, Aartman IH, Parsa A, Tahmaseb A, Wismeijer D (2017) Implant Mandibular overdentures retained by immediately loaded implants: a 1-Year randomized Trial comparing the clinical and radiographic outcomes between Mini Dental implants and Standard-Sized implants. Int J Oral Maxillofac Implants 32:1377–1388. 10.11607/jomi.5981 29140382 10.11607/jomi.5981

[71] Zygogiannis K, Wismeijer D, Parsa A (2016) A pilot study on Mandibular overdentures retained by Mini Dental implants: marginal bone level changes and patient-based ratings of clinical outcome. Int J Oral Maxillofac Implants 31:1171–1178. 10.11607/jomi.4339 27632275 10.11607/jomi.4339

[72] Chrcanovic BR, Kisch J, Albrektsson T, Wennerberg A (2016) Factors influencing early Dental Implant failures. J Dent Res 95:995–1002. 10.1177/0022034516646098 27146701 10.1177/0022034516646098

[73] Chrcanovic BR, Kisch J, Albrektsson T, Wennerberg A (2018) A retrospective study on clinical and radiological outcomes of oral implants in patients followed up for a minimum of 20 years. Clin Implant Dent Relat Res 20:199–207. 10.1111/cid.12571 29210186 10.1111/cid.12571

[74] Ivanoff CJ, Sennerby L, Johansson C, Rangert B, Lekholm U (1997) Influence of implant diameters on the integration of screw implants. An experimental study in rabbits. Int J Oral Maxillofac Surg 26:141–148 9151173 10.1016/s0901-5027(05)80837-9

[75] Zinsli B, Sägesser T, Mericske E, Mericske-Stern R (2004) Clinical evaluation of small-diameter ITI implants: a prospective study. Int J Oral Maxillofac Implants 19:92–99 14982361

[76] Javed F, Ahmed HB, Crespi R, Romanos GE (2013) Role of primary stability for successful osseointegration of dental implants: factors of influence and evaluation. Interventional Med Appl Sci 5:162–167. 10.1556/imas.5.2013.4.3 10.1556/IMAS.5.2013.4.3 PMC3873594 24381734

[77] Chrcanovic BR, Albrektsson T, Wennerberg A (2017) Bone Quality and Quantity and Dental Implant failure: a systematic review and Meta-analysis. Int J Prosthodont 30:219–237. 10.11607/ijp.5142 28319206 10.11607/ijp.5142

[78] Ghiasi P, Ahlgren C, Larsson C, Chrcanovic BR (2021) Implant and prosthesis failure rates with implant-supported maxillary overdentures: a systematic review. Int J Prosthodont 34:482–491. 10.11607/ijp.6905 33625390 10.11607/ijp.6905

[79] Chung S, McCullagh A, Irinakis T (2011) Immediate loading in the maxillary arch: evidence-based guidelines to improve success rates: a review. J Oral Implantol 37:610–621. 10.1563/aaid-d-joi-10-00058.1 22004059 10.1563/AAID-D-JOI-10-00058.1

[80] Lioubavina-Hack N, Lang NP, Karring T (2006) Significance of primary stability for osseointegration of dental implants. Clinical oral implants research 17:244– 50. 10.1111/j.1600-0501.2005.01201.x 10.1111/j.1600-0501.2005.01201.x 16672018

[81] Trisi P, Perfetti G, Baldoni E, Berardi D, Colagiovanni M, Scogna G (2009) Implant micromotion is related to peak insertion torque and bone density. Clinical oral implants research 20:467– 71. 10.1111/j.1600-0501.2008.01679.x 10.1111/j.1600-0501.2008.01679.x 19522976

[82] Stocchero M, Jinno Y, Toia M, Ahmad M, Galli S, Papia E, Herath M, Becktor JP (2023) Effect of Drilling Preparation on immediately loaded implants: an in vivo study in Sheep. Int J Oral Maxillofac Implants 38:607–618. 10.11607/jomi.9949 37279224 10.11607/jomi.9949

[83] Jiang X, Zhou W, Wu Y, Wang F (2021) Clinical outcomes of Immediate Implant Loading with fixed prostheses in Edentulous Maxillae: a systematic review. Int J Oral Maxillofac Implants 36:503–519. 10.11607/jomi.8509 34115065 10.11607/jomi.8509

[84] Liu J, Pan S, Dong J, Mo Z, Fan Y, Feng H (2013) Influence of implant number on the biomechanical behaviour of mandibular implant-retained/supported overdentures: a three-dimensional finite element analysis. J Dent 41:241–249. 10.1016/j.jdent.2012.11.008 23160036 10.1016/j.jdent.2012.11.008

[85] Domingo KB, Burgess JO, Litaker MS, McCracken MS (2013) Strength comparison of four techniques to secure implant attachment housings to complete dentures. J Prosthet Dent 110:8–13. 10.1016/s0022-3913(13)60332-7 23849608 10.1016/S0022-3913(13)60332-7

[86] Kern M, Att W, Fritzer E, Kappel S, Luthardt RG, Mundt T, Reissmann DR, Rädel M, Stiesch M, Wolfart S, Passia N (2018) Survival and complications of single Dental implants in the Edentulous Mandible following Immediate or delayed loading: a Randomized Controlled Clinical Trial. J Dent Res 97:163–170. 10.1177/0022034517736063 29045800 10.1177/0022034517736063 PMC6029143

[87] Chrcanovic BR, Ghiasi P, Kisch J, Lindh L, Larsson C (2020) Retrospective study comparing the clinical outcomes of bar-clip and ball attachment implant-supported overdentures. J Oral Sci 62:397–401. 10.2334/josnusd.19-0412 32848099 10.2334/josnusd.19-0412

[88] De Bruyn H, Raes S, Ostman P, Cosyn J (2014) Immediate loading in partially and completely edentulous jaws: a review of the literature with clinical guidelines. Periodontol 2000 66(153–87). 10.1111/prd.12040 10.1111/prd.12040 25123767

[89] Attard NJ, Diacono M (2010) Early loading of fixture original implants with mandibular overdentures–a preliminary report on a prospective study. Int J Prosthodont 23:507–512 21209984

[90] Matsushita Y, Kitoh M, Mizuta K, Ikeda H, Suetsugu T (1990) Two-dimensional FEM analysis of hydroxyapatite implants: diameter effects on stress distribution. J Oral Implantol 16:6–11 2074593

[91] Aunmeungtong W, Khongkhunthian P, Rungsiyakull P (2016) Stress and strain distribution in three different mini dental implant designs using in implant retained overdenture: a finite element analysis study. ORAL Implantology 9:202–212. 10.11138/orl/2016.9.4.202 28042449 10.11138/orl/2016.9.4.202 PMC5159942

[92] Hasan I, Heinemann F, Aitlahrach M, Bourauel C (2010) Biomechanical finite element analysis of small diameter and short dental implant. Biomedizinische Technik Biomedical Eng 55:341–350. 10.1515/bmt.2010.049 10.1515/BMT.2010.049 21028950

[93] Wennerberg A, Albrektsson T, Chrcanovic B (2018) Long-term clinical outcome of implants with different surface modifications. Eur J Oral Implantol 11(Suppl 1):S123–s136 30109304

[94] Al Ansari Y, Shahwan H, Chrcanovic BR (2022) Diabetes Mellitus and Dental implants: a systematic review and Meta-analysis. Mater (Basel Switzerland) 15:3227. 10.3390/ma15093227 10.3390/ma15093227 PMC9105616 35591561

[95] Chrcanovic BR, Kisch J, Albrektsson T, Wennerberg A (2017) Is the intake of selective serotonin reuptake inhibitors associated with an increased risk of dental implant failure? Int J Oral Maxillofac Surg 46:782–788. 10.1016/j.ijom.2017.01.016 28222946 10.1016/j.ijom.2017.01.016

[96] Chrcanovic BR, Kisch J, Albrektsson T, Wennerberg A (2017) Intake of Proton Pump inhibitors is Associated with an increased risk of Dental Implant failure. Int J Oral Maxillofac Implants 32:1097–1102. 10.11607/jomi.5662 28632255 10.11607/jomi.5662

[97] Häggman-Henrikson B, Ali D, Aljamal M, Chrcanovic BR (2023) Bruxism and dental implants: a systematic review and meta-analysis. J Rehabil. 10.1111/joor.13567 10.1111/joor.13567 37589382

[98] Mustapha AD, Salame Z, Chrcanovic BR (2021) Smoking and Dental implants: a systematic review and Meta-analysis. Med (Kaunas Lithuania) 58:39. 10.3390/medicina58010039 10.3390/medicina58010039 PMC8780868 35056347

[99] Sulaiman N, Fadhul F, Chrcanovic BR (2023) Bisphosphonates and Dental implants: a systematic review and Meta-analysis. Mater (Basel Switzerland) 16:6078. 10.3390/ma16186078 10.3390/ma16186078 PMC10532755 37763356

