# Supplementary material for: Clinical and radiographic outcomes of mini-implant-retained maxillary and mandibular overdentures: a systematic review and meta-analysis
Source: Clin Oral Investig. 2025 Mar 1;29(3):164. doi: 10.1007/s00784-025-06242-3 (PMC11872979; doi:10.1007/s00784-025-06242-3)
Supplement: Supplementary file 1 — Supplementary Material 1 [file 784_2025_6242_MOESM1_ESM.docx]

**SUPPLEMENTARY MATERIAL**

**Clinical and radiographic outcomes of** **mini-implant-retained overdentures: a systematic review and meta-analysis**

1. **List of excluded articles**
2. **Table S1. Detailed data of the included studies**
3. **Table S2. Quality assessment of the included studies, according to the National Institutes of Health (NIH)**
4. **Table S3. Quality assessment of the included case reports, according to the CARE guidelines**

**a. Excluded studies**

1. Abou-Ayash S, Enkling N, Srinivasan M, Haueter M, Worni A, Schimmel M. Evolution of in vivo assessed retention forces in one-piece mini dental implant-retained mandibular overdentures: 5-Year follow-up of a prospective clinical trial. Clin Implant Dent Relat Res. 2019 Oct;21(5):968-976.
2. Agusto M, Kordusky B, Bryington MS, Salman A, Schincaglia GP. Immediate Loading of Four Guided Unsplinted Narrow Implants Supporting a Maxillary Overdenture: A Minimally Invasive Approach. Clin Adv Periodontics. 2022 Jun;12(2):118-123.
3. Al Jaghsi A, Mundt T. Upgrading removable denture design by using strategic implants: A case report. Ann Anat. 2023 Jan;245:152002.
4. Al-Nawas B, Brägger U, Meijer HJ, Naert I, Persson R, Perucchi A, Quirynen M, Raghoebar GM, Reichert TE, Romeo E, Santing HJ, Schimmel M, Storelli S, ten Bruggenkate C, Vandekerckhove B, Wagner W, Wismeijer D, Müller F. A double-blind randomized controlled trial (RCT) of Titanium-Zirconium versus Titanium Grade IV small-diameter bone level implants in edentulous mandibles--results from a 1-year observation period. Clin Implant Dent Relat Res. 2012 Dec;14(6):896-904.
5. Alshenaiber R, Barclay C, Silikas N. The Effect of Mini Dental Implant Number on Mandibular Overdenture Retention and Attachment Wear. Biomed Res Int. 2023 Apr 30;2023:7099761.
6. Altuna P, Fernández-Villar S, Barroso-Panella A, Ortiz-Puigpelat O, Hernández-Alfaro F, Nart J. Narrow diameter titanium-zirconium tissue-level implants supporting multi-unit FDPs in the anterior area: A 5-year prospective study. Clin Oral Implants Res. 2023 Jul;34(7):751-767.
7. Anitua E, Errazquin JM, de Pedro J, Barrio P, Begoña L, Orive G. Clinical evaluation of Tiny® 2.5- and 3.0-mm narrow-diameter implants as definitive implants in different clinical situations: a retrospective cohort study. Eur J Oral Implantol. 2010 Winter;3(4):315-22.
8. Ashmawy TM, El Talawy DB, Shaheen NH. Effect of mini-implant-supported mandibular overdentures on electromyographic activity of the masseter muscle during chewing of hard and soft food. Quintessence Int. 2014 Sep;45(8):663-71.
9. Aunmeungtong W, Khongkhunthian P, Rungsiyakull P. Stress and strain distribution in three different mini dental implant designs using in implant retained overdenture: a finite element analysis study. Oral Implantol (Rome). 2016 Nov 16;9(4):202-212.
10. Aunmeungtong W, Kumchai T, Strietzel FP, Reichart PA, Khongkhunthian P. Comparative Clinical Study of Conventional Dental Implants and Mini Dental Implants for Mandibular Overdentures: A Randomized Clinical Trial. Clin Implant Dent Relat Res. 2017 Apr;19(2):328-340.
11. Bidra AS, Almas K. Mini implants for definitive prosthodontic treatment: a systematic review. J Prosthet Dent. 2013 Mar;109(3):156-64.
12. Bielemann AM, Marcello-Machado RM, Schuster AJ, Chagas Júnior OL, Del Bel Cury AA, Faot F. Healing differences in narrow diameter implants submitted to immediate and conventional loading in mandibular overdentures: A randomized clinical trial. J Periodontal Res. 2019 Jun;54(3):241-250.
13. Borges GA, Presotto AGC, Caldas RA, Pisani MX, Mesquita MF. Is one dental mini-implant biomechanically appropriate for the retention of a mandibular overdenture? A comparison with Morse taper and external hexagon platforms. J Prosthet Dent. 2021 Mar;125(3):491-499.
14. Bulard RA, Vance JB. Multi-clinic evaluation using mini-dental implants for long-term denture stabilization: a preliminary biometric evaluation. Compend Contin Educ Dent. 2005 Dec;26(12):892-7.
15. Cezar Pomini M, Postiglione Buhrer Samra A, Regina Fischborn A, Antônio Kozlowski Junior V, Bucholdz Teixeira Alves F. The use of mini-implants for provisional prosthetic rehabilitation in growing patients: a critical review. J Prosthodont Res. 2021 Feb 24;65(1):19-24.
16. Chang SH, Huang SR, Huang SF, Lin CL. Mechanical response comparison in an implant overdenture retained by ball attachments on conventional regular and mini dental implants: a finite element analysis. Comput Methods Biomech Biomed Engin. 2016;19(8):911-21.
17. Chaves CA, Souza RF, Cunha TR, Vecchia MP, Ribeiro AB, Bruniera JF, Silva-Sousa YT. Preliminary In Vitro Study on O-Ring Wear in Mini-Implant-Retained Overdentures. Int J Prosthodont. 2016 Jul-Aug;29(4):357-9.
18. Chen B, Lin Y, di P, Li JH, Qiu LX. [Preliminary clinical study of microimplants in the restoration of edentulous jaws]. Zhonghua Kou Qiang Yi Xue Za Zhi. 2006 Apr;41(4):200-2.
19. Cho SC, Froum S, Tai CH, Cho YS, Elian N, Tarnow DP. Immediate loading of narrow-diameter implants with overdentures in severely atrophic mandibles. Pract Proced Aesthet Dent. 2007 Apr;19(3):167-74.
20. Cordaro L, Torsello F, Mirisola di Torresanto V, Baricevic M. Rehabilitation of an edentulous atrophic maxilla with four unsplinted narrow diameter titanium-zirconium implants supporting an overdenture. Quintessence Int. 2013 Jan;44(1):37-43.
21. Ćorić A, Kovačić I, Kiršić SP, Čelebić A. Are Mini Dental Implants Suitable for Support of Crowns or Small Bridges in the Mandibular Incisor Region? A 5-year Longitudinal Study. J Oral Maxillofac Surg. 2022 Nov;80(11):1811-1826.
22. da Costa Valente ML, Macedo AP, Dos Reis AC. Stress Distribution Analysis of Novel Dental Mini-Implant Designs to Support Overdenture Prosthesis. J Oral Implantol. 2022 Apr 1;48(2):79-83.
23. de Oliveira Rigotti RL, Tardelli JDC, Dos Reis AC, da Valente MLC. Influence of dental implant/mini-implant design on stress distribution in overdentures: a systematic review. Oral Maxillofac Surg. 2023 Sep 4.
24. Degidi M, Nardi D, Piattelli A. Immediate restoration of small-diameter implants in cases of partial posterior edentulism: a 4-year case series. J Periodontol. 2009 Jun;80(6):1006-12.
25. Della Vecchia MP, Leles CR, Cunha TR, Ribeiro AB, Sorgini DB, Muglia VA, Reis AC, Albuquerque RF Jr, de Souza RF. Mini-Implants for Mandibular Overdentures: Cost-Effectiveness Analysis alongside a Randomized Trial. JDR Clin Trans Res. 2018 Jan;3(1):47-56.
26. Dhaliwal JS, Albuquerque RF Jr, Fakhry A, Kaur S, Feine JS. Customized SmartPeg for measurement of resonance frequency of mini dental implants. Int J Implant Dent. 2017 Dec;3(1):4.
27. Dörsam I, Bauroth A, Keilig L, Bourauel C, Heinemann F. Definition of a drilling protocol for mini dental implants in different bone qualities. Ann Anat. 2020 Sep;231:151511.
28. El-Sheikh AM, Shihabuddin OF, Ghoraba SM. Two versus three narrow-diameter implants with locator attachments supporting mandibular overdentures: a two-year prospective study. Int J Dent. 2012;2012:285684.
29. Elsyad MA, Gebreel AA, Fouad MM, Elshoukouki AH. The clinical and radiographic outcome of immediately loaded mini implants supporting a mandibular overdenture. A 3-year prospective study. J Oral Rehabil. 2011 Nov;38(11):827-34.
30. Enkling N, Haueter M, Worni A, Müller F, Leles CR, Schimmel M. A prospective cohort study on survival and success of one-piece mini-implants with associated changes in oral function: Five-year outcomes. Clin Oral Implants Res. 2019 Jun;30(6):570-577.
31. Enkling N, Saftig M, Worni A, Mericske-Stern R, Schimmel M. Chewing efficiency, bite force and oral health-related quality of life with narrow diameter implants - a prospective clinical study: results after one year. Clin Oral Implants Res. 2017 Apr;28(4):476-482.
32. Erwood I. Denture stabilization with small-diameter implants. Dent Today. 2010 Jul;29(7):116-8.
33. Fatalla AA, Song K, Cao YG. New mini dental implant attachments versus O-ring attachment after cyclic aging: Analysis of retention strength and gap space. J Huazhong Univ Sci Technolog Med Sci. 2017 Jun;37(3):419-424.
34. Flanagan D. Mini Implants Supporting Fixed Partial Dentures in the Posterior Mandible: A Retrospective. J Oral Implantol. 2015 Aug;41(4):e138-43.
35. Fonteyne E, Van Doorne L, Becue L, Matthys C, Bronckhorst E, De Bruyn H. Speech evaluation during maxillary mini-dental implant overdenture treatment: A prospective study. J Oral Rehabil. 2019 Dec;46(12):1151-1160.
36. Garhnayak M, Garhnayak L, Dev S, Kar AK, Mohapatra A. Prosthodontic management of flat mandibular ridge by mini implant supported over denture. J Clin Diagn Res. 2014 Jul;8(7):ZD19-21.
37. Giannakopoulos NN, Ariaans K, Eberhard L, Klotz AL, Oh K, Kappel S. Immediate and delayed loading of two-piece reduced-diameter implants with locator-analog attachments in edentulous mandibles: One-year results from a randomized clinical trial examining clinical outcome and patient expectation. Clin Implant Dent Relat Res. 2017 Aug;19(4):643-653.
38. Goiato MC, Sônego MV, Pellizzer EP, Gomes JML, da Silva EVF, Dos Santos DM. Clinical outcome of removable prostheses supported by mini dental implants. A systematic review. Acta Odontol Scand. 2018 Nov;76(8):628-637.
39. Guo Y, Kono K, Suzuki Y, Ohkubo C, Zeng JY, Zhang J. Influence of marginal bone resorption on two mini implant-retained mandibular overdenture: An in vitro study. J Adv Prosthodont. 2021 Feb;13(1):55-64.
40. Hallman M. A prospective study of treatment of severely resorbed maxillae with narrow nonsubmerged implants: results after 1 year of loading. Int J Oral Maxillofac Implants. 2001 Sep-Oct;16(5):731-6.
41. Hasan I, Madarlis C, Keilig L, Dirk C, Weber A, Bourauel C, Heinemann F. Changes in biting forces with implant-supported overdenture in the lower jaw: A comparison between conventional and mini implants in a pilot study. Ann Anat. 2016 Nov;208:116-122.
42. Huard C, Bessadet M, Nicolas E, Veyrune JL. Geriatric slim implants for complete denture wearers: clinical aspects and perspectives. Clin Cosmet Investig Dent. 2013 Aug 28;5:63-8.
43. Ishida Y, Kumar HSK, Goto T, Watanabe M, Wigianto R, Ichikawa T. Magnet-Retained Two-Mini-Implant Overdenture: Clinical and Mechanical Consideration. Dent J (Basel). 2016 Oct 10;4(4):35.
44. Jackson BJ. Small-diameter implants: a treatment consideration for the maxillary edentulous patient. Dent Today. 2013 Nov;32(11):86, 88, 90-2; quiz 93.
45. Janev E, Janeva N, Peeva-Petreska M, Mitic K. Therapeutic Challenge in a Severely Atrophic Mandible. Open Access Maced J Med Sci. 2018 Mar 10;6(3):564-567.
46. Jia-Mahasap W, Rungsiyakull C, Bumrungsiri W, Sirisereephap N, Rungsiyakull P. Effect of Number and Location on Stress Distribution of Mini Dental Implant-Assisted Mandibular Kennedy Class I Removable Partial Denture: Three-Dimensional Finite Element Analysis. Int J Dent. 2022 Mar 26;2022:4825177.
47. Jofre J, Castiglioni X, Lobos CA. Influence of minimally invasive implant-retained overdenture on patients' quality of life: a randomized clinical trial. Clin Oral Implants Res. 2013 Oct;24(10):1173-7.
48. Jofre J, Cendoya P, Munoz P. Effect of splinting mini-implants on marginal bone loss: a biomechanical model and clinical randomized study with mandibular overdentures. Int J Oral Maxillofac Implants. 2010 Nov-Dec;25(6):1137-44.
49. Jofré J, Hamada T, Nishimura M, Klattenhoff C. The effect of maximum bite force on marginal bone loss of mini-implants supporting a mandibular overdenture: a randomized controlled trial. Clin Oral Implants Res. 2010 Feb;21(2):243-9.
50. Kanazawa M, Feine J, Esfandiari S. Clinical guidelines and procedures for provision of mandibular overdentures on 4 mini-dental implants. J Prosthet Dent. 2017 Jan;117(1):22-27.
51. Kobayashi M, Ohkubo C, Suzuki Y, Aoki T, Sato J, Hosoi T. Retentive force of O-ring attachment to use Immediate Provisional Implant (IPI)-retained overdenture. Eur J Prosthodont Restor Dent. 2005 Dec;13(4):147-9.
52. LaBarre EE, Ahlstrom RH, Noble WH. Narrow diameter implants for mandibular denture retention. J Calif Dent Assoc. 2008 Apr;36(4):283-6.
53. Lee E, Shin SY. The influence of the number and the type of magnetic attachment on the retention of mandibular mini implant overdenture. J Adv Prosthodont. 2017 Feb;9(1):14-21.
54. Leles CR, de Paula MS, Curado TFF, Silva JR, Leles JLR, McKenna G, Schimmel M. Flapped versus flapless surgery and delayed versus immediate loading for a four mini implant mandibular overdenture: A RCT on post-surgical symptoms and short-term clinical outcomes. Clin Oral Implants Res. 2022 Sep;33(9):953-964.
55. Leles CR, Leles JLR, Curado TFF, Silva JR, Nascimento LN, de Paula MS, Maniewicz S, Schimmel M, McKenna G. Mandibular bone characteristics, drilling protocols, and final insertion torque for titanium-zirconium mini-implants for overdentures: A cross-sectional analysis. Clin Implant Dent Relat Res. 2023 Apr;25(2):426-434.
56. Leles CR, Leles JLR, Curado TFF, Silva JR, Nascimento LN, de Paula MS, Maniewicz S, Schimmel M, McKenna G. Mandibular bone characteristics, drilling protocols, and final insertion torque for titanium-zirconium mini-implants for overdentures: A cross-sectional analysis. Clin Implant Dent Relat Res. 2023 Apr;25(2):426-434.
57. Lemos CA, Verri FR, Batista VE, Júnior JF, Mello CC, Pellizzer EP. Complete overdentures retained by mini implants: A systematic review. J Dent. 2017 Feb;57:4-13.
58. Liao XL, Pan SX, Feng HL, Kang YF, Peng D. [Effect of early-loaded small diameter implants retained mandibular overdentures on masticatory performance]. Beijing Da Xue Xue Bao Yi Xue Ban. 2013 Oct 18;45(5):798-802.
59. Mangano F, Pozzi-Taubert S, Zecca PA, Luongo G, Sammons RL, Mangano C. Immediate restoration of fixed partial prostheses supported by one-piece narrow-diameter selective laser sintering implants: a 2-year prospective study in the posterior jaws of 16 patients. Implant Dent. 2013 Aug;22(4):388-93.
60. Mangano FG, Caprioglio A, Levrini L, Farronato D, Zecca PA, Mangano C. Immediate loading of mandibular overdentures supported by one-piece, direct metal laser sintering mini-implants: a short-term prospective clinical study. J Periodontol. 2015 Feb;86(2):192-200.
61. Marcello-Machado RM, Faot F, Schuster AJ, Bielemann AM, Chagas Júnior OL, Del Bel Cury AA. One-year clinical outcomes of locking taper Equator attachments retaining mandibular overdentures to narrow diameter implants. Clin Implant Dent Relat Res. 2018 Aug;20(4):483-492.
62. Marcello-Machado RM, Faot F, Schuster AJ, Bielemann AM, Nascimento GG, Del Bel Cury AA. Mapping of inflammatory biomarkers in the peri-implant crevicular fluid before and after the occlusal loading of narrow diameter implants. Clin Oral Investig. 2020 Mar;24(3):1311-1320.
63. Marcello-Machado RM, Faot F, Schuster AJ, Bielemann AM, Nascimento GG, Del Bel Cury AA. How fast can treatment with overdentures improve the masticatory function and OHRQoL of atrophic edentulous patients? A 1-year longitudinal clinical study. Clin Oral Implants Res. 2018 Feb;29(2):215-226.
64. Marcello-Machado RM, Faot F, Schuster AJ, Nascimento GG, Del Bel Cury AA. Mini-implants and narrow diameter implants as mandibular overdenture retainers: A systematic review and meta-analysis of clinical and radiographic outcomes. J Oral Rehabil. 2018 Feb;45(2):161-183.
65. Meleşcanu Imre M, Preoteasa E, Țâncu A, Preoteasa CT. Imaging technique for the complete edentulous patient treated conventionally or with mini implant overdenture. J Med Life. 2013 Mar 15;6(1):86-92.
66. Mifsud DP, Cortes ARG, Attard NJ. Patient-based outcomes with conventional or mini-implants immediately loaded with locator-retained mandibular overdentures: A cohort study. Clin Implant Dent Relat Res. 2020 Dec;22(6):723-729.
67. Misch K, Neiva R. Small-diameter implants for optimal stabilization of implant-supported overdentures. Pract Proced Aesthet Dent. 2007 Aug;19(7):428-31.
68. Müller F, Al-Nawas B, Storelli S, Quirynen M, Hicklin S, Castro-Laza J, Bassetti R, Schimmel M; Roxolid Study Group. Small-diameter titanium grade IV and titanium-zirconium implants in edentulous mandibles: five-year results from a double-blind, randomized controlled trial. BMC Oral Health. 2015 Oct 12;15(1):123.
69. Mundt T, Al Jaghsi A, Schwahn B, Hilgert J, Lucas C, Biffar R, Schwahn C, Heinemann F. Immediate versus delayed loading of strategic mini dental implants for the stabilization of partial removable dental prostheses: a patient cluster randomized, parallel-group 3-year trial. BMC Oral Health. 2016 Jul 30;17(1):30.
70. Mundt T, Heinemann F, Müller J, Schwahn C, Al Jaghsi A. Survival and stability of strategic mini-implants with immediate or delayed loading under removable partial dentures: a 3-year randomized controlled clinical trial. Clin Oral Investig. 2023 Apr;27(4):1767-1779.
71. Mundt T, Schwahn C, Biffar R, Heinemann F. Changes in Bone Levels Around Mini-Implants in Edentulous Arches. Int J Oral Maxillofac Implants. 2015 Sep-Oct;30(5):1149-55.
72. Omran M, Abdelhamid A, Elkarargy A, Sallom M. Mini-implant overdenture versus conventional implant overdenture (A radiographic and clinical assessments). J Am Sci 2013;9(9):89-97.
73. Park JH, Lee JY, Shin SW. Treatment Outcomes for Mandibular Mini-Implant-Retained Overdentures: A Systematic Review. Int J Prosthodont. 2017 May/June;30(3):269–276.
74. Park JH, Shin SW, Lee JY. Two-Step Immediate Loading of Mandibular Overdentures Retained by Mini-implants: A Prospective Clinical Study. Int J Prosthodont. 2018 September/October;31(5):446–450.
75. Park JH, Shin SW, Lee JY. Narrow-diameter versus regular-diameter dental implants for mandibular overdentures: A systematic review and meta-analysis. J Prosthodont. 2023 Jun 27.
76. Patel PB. Maxillary small-diameter implant-retained overdentures. Dent Today. 2013 Jul;32(7):101-3.
77. Patel PB. Utilizing Angled O-Ball Narrow-Diameter Implants to Solve the Restorative Challenge Posed by Alveolar Resorption: A Case Report. Compend Contin Educ Dent. 2015 Sep;36(8):607-11.
78. Patil PG, Seow LL, Uddanwadikar R, Ukey PD. Biomechanical behavior of mandibular overdenture retained by two standard implants or 2 mini implants: A 3-dimensional finite element analysis. J Prosthet Dent. 2021 Jan;125(1):138.e1-138.e8.
79. Peršić S, Ćelić R, Vojvodić D, Petričević N, Kranjčić J, Zlatarić DK, Čelebić A. Oral Health-Related Quality of Life in Different Types of Mandibular Implant Overdentures in Function Longer Than 3 Years. Int J Prosthodont. 2016 Jan-Feb;29(1):28-30.
80. Pisani MX, Presotto AGC, Mesquita MF, Barão VAR, Kemmoku DT, Del Bel Cury AA. Biomechanical behavior of 2-implant- and single-implant-retained mandibular overdentures with conventional or mini implants. J Prosthet Dent. 2018 Sep;120(3):421-430.
81. Preoteasa E, Marin M, Imre M, Lerner H, Preoteasa CT. Patients' satisfaction with conventional dentures and mini implant anchored overdentures. Rev Med Chir Soc Med Nat Iasi. 2012 Jan-Mar;116(1):310-6.
82. Preoteasa E, Meleşcanu-Imre M, Preoteasa CT, Marin M, Lerner H. Aspects of oral morphology as decision factors in mini-implant supported overdenture. Rom J Morphol Embryol. 2010;51(2):309-14.
83. Quirynen M, Al-Nawas B, Meijer HJ, Razavi A, Reichert TE, Schimmel M, Storelli S, Romeo E; Roxolid Study Group. Small-diameter titanium Grade IV and titanium-zirconium implants in edentulous mandibles: three-year results from a double-blind, randomized controlled trial. Clin Oral Implants Res. 2015 Jul;26(7):831-40.
84. Reis R, Nicolau P, Calha N, Messias A, Guerra F. Immediate versus early loading protocols of titanium-zirconium narrow-diameter implants for mandibular overdentures in edentulous patients: 1-year results from a randomized controlled trial. Clin Oral Implants Res. 2019 Oct;30(10):953-961.
85. Reissmann DR, Enkling N, Moazzin R, Haueter M, Worni A, Schimmel M. Long-term changes in oral health-related quality of life over a period of 5 years in patients treated with narrow diameter implants: A prospective clinical study. J Dent. 2018 Aug;75:84-90.
86. Ribeiro AB, Della Vecchia MP, Cunha TR, Sorgini DB, Dos Reis AC, Muglia VA, de Albuquerque RF Jr, de Souza RF. Short-term post-operative pain and discomfort following insertion of mini-implants for retaining mandibular overdentures: a randomized controlled trial. J Oral Rehabil. 2015 Aug;42(8):605-14.
87. Roy S, Maji S, Paul R, Bhattacharyya J, Goel P. A comparison of cost and cost-effectiveness analysis of two- implant-retained overdentures versus other removable prosthodontic treatment options for edentulous mandible: A systematic review. J Indian Prosthodont Soc. 2020 Apr-Jun;20(2):162-170.
88. Šćepanović M, Todorović A, Marković A, Patrnogić V, Miličić B, Moufti AM, Mišić T. Immediately loaded mini dental implants as overdenture retainers: 1-Year cohort study of implant stability and peri-implant marginal bone level. Ann Anat. 2015 May;199:85-91.
89. Scherer MD, Ingel AP, Rathi N. Flapped or flapless surgery for narrow-diameter implant placement for overdentures: advantages, disadvantages, indications, and clinical rationale. Int J Periodontics Restorative Dent. 2014;34 Suppl 3:s89-95.
90. Scherer MD. Narrow-Diameter Overdenture Implants: A Sensible Option for Growing a Practice. Compend Contin Educ Dent. 2015 Jul-Aug;36(7):460-1.
91. Schuster AJ, da Rosa Possebon AP, Schinestsck AR, Chagas-Júnior OL, Faot F. Circumferential bone level and bone remodeling in the posterior mandible of edentulous mandibular overdenture wearers: influence of mandibular bone atrophy in a 3-year cohort study. Clin Oral Investig. 2022 Mar;26(3):3119-3130.
92. Schuster AJ, Possebon APDR, Bielemann AM, Chagas-Júnior OL, Faot F. Effect of mandibular residual ridge regularization on peri-implant wound healing when narrow diameter implants are used as overdenture retainers. J Prosthet Dent. 2022 Oct;128(4):648-655.
93. Schuster AJ, Possebon APDR, Schinestsck AR, Chagas-Júnior OL, Faot F. Effect of mandibular bone atrophy on maxillary and mandibular bone remodeling and quality of life with an implant-retained mandibular overdenture after 3 years. J Prosthet Dent. 2023 Aug;130(2):220-228.
94. Singh RD, Ramashanker, Chand P. Management of atrophic mandibular ridge with mini dental implant system. Natl J Maxillofac Surg. 2010 Jul;1(2):176-8.
95. Sivaramakrishnan G, Sridharan K. Comparison of patient satisfaction with mini-implant versus standard diameter implant overdentures: a systematic review and meta-analysis of randomized controlled trials. Int J Implant Dent. 2017 Dec;3(1):29.
96. Soğancı G, Yazıcıoğlu H. Evaluation of Stress Distribution of Mini Dental Implant-Supported Overdentures in Complete Cleft Palate Models: A Three-Dimensional Finite Element Analysis Study. Cleft Palate Craniofac J. 2016 Jan;53(1):73-83.
97. Sohrabi K, Mushantat A, Esfandiari S, Feine J. How successful are small-diameter implants? A literature review. Clin Oral Implants Res. 2012 May;23(5):515-25.
98. Sussman HI, Goodridge OF. Use of SIG device to accurately place permanent miniature dental implants to retain mandibular overdenture. A case report. N Y State Dent J. 2006 Aug-Sep;72(5):34-8.
99. Takagaki K, Gonda T, Maeda Y. Lateral forces exerted through ball or bar attachments in relation to the inclination of mini-implant underneath overdentures: in vitro study. Clin Oral Implants Res. 2015 Sep;26(9):1060-3.
100. Takagaki K, Gonda T, Maeda Y. Number and Location of Mini-Implants Retaining a Mandibular Overdenture to Resist Lateral Forces: A Preliminary In Vitro Study. Int J Prosthodont. 2017 May/June;30(3):248–250.
101. Threeburuth W, Aunmeungtong W, Khongkhunthian P. Comparison of immediate-load mini dental implants and conventional-size dental implants to retain mandibular Kennedy class I removable partial dentures: A randomized clinical trial. Clin Implant Dent Relat Res. 2018 Oct;20(5):785-792.
102. Trang BNH, Kanazawa M, Murakami N, Wakabayashi N, Hada T, Sahaprom N, Komagamine Y, Minakuchi S. Stress distribution of one-piece and two-piece mini-Implant overdentures with various attachment systems and diameters: A finite element analysis. J Prosthodont Res. 2022 Nov 12.
103. Valente MLC, Bolfarini C, de Oliveira DP, Dos Reis AC. Dental mini-implant designs to support overdentures: Development, biomechanical evaluation, and 3D digital image correlation. J Prosthet Dent. 2022 Oct;128(4):754-763.
104. Valente MLC, Shimano MVW, Agnelli JAM, Dos Reis AC. Retention force and deformation of an innovative attachment model for mini-implant-retained overdentures. J Prosthet Dent. 2019 Jan;121(1):129-134.
105. Van Doorne L, De Backer B, Matthys C, De Bruyn H, Vandeweghe S. Comparing Masticatory Performance of Maxillary Mini Dental Implant Overdentures, Complete Removable Dentures and Dentate Subjects. J Clin Med. 2021 Oct 27;10(21):5006.
106. Van Doorne L, De Kock L, De Moor A, Shtino R, Bronkhorst E, Meijer G, De Bruyn H. Flaplessly placed 2.4-mm mini-implants for maxillary overdentures: a prospective multicentre clinical cohort study. Int J Oral Maxillofac Surg. 2020 Mar;49(3):384-391.
107. Van Doorne L, Fonteyne E, Matthys C, Bronkhorst E, Meijer G, De Bruyn H. "Longitudinal Oral Health-Related Quality of Life in maxillary mini dental implant overdentures after 3 years in function". Clin Oral Implants Res. 2021 Jan;32(1):23-36.
108. Vi S, Pham D, Du YYM, Arora H, Tadakamadla SK. Mini-Implant-Retained Overdentures for the Rehabilitation of Completely Edentulous Maxillae: A Systematic Review and Meta-Analysis. Int J Environ Res Public Health. 2021 Apr 20;18(8):4377.
109. Wang B, Ho KS, Neo TK, Cheng AC. Mini-dental implants for definitive prosthesis retention - A synopsis of the current evidence. Singapore Dent J. 2019 Dec;39(1):1-9.
110. Warin P, Rungsiyakull P, Rungsiyakull C, Khongkhunthian P. Effects of different numbers of mini-dental implants on alveolar ridge strain distribution under mandibular implant-retained overdentures. J Prosthodont Res. 2018 Jan;62(1):35-43.
111. Worni A, Hicklin SP, Mericske-Stern R, Enkling N. Performance and marginal bone level alteration around immediately loaded narrow-diameter implants. A prospective clinical study: Results after 1 year. Quintessence Int. 2018;49(4):267-276.
112. Zinsli B, Sägesser T, Mericske E, Mericske-Stern R. Clinical evaluation of small-diameter ITI implants: a prospective study. Int J Oral Maxillofac Implants. 2004 Jan-Feb;19(1):92-9.
113. Zweers J, van Doornik A, Hogendorf EA, Quirynen M, Van der Weijden GA. Clinical and radiographic evaluation of narrow- vs. regular-diameter dental implants: a 3-year follow-up. A retrospective study. Clin Oral Implants Res. 2015 Feb;26(2):149-56.
114. Zygogiannis K, Aartman IH, Wismeijer D. Implant Mandibular Overdentures Retained by Immediately Loaded Implants: A 1-Year Randomized Trial Comparing Patient-Based Outcomes Between Mini Dental Implants and Standard-Sized Implants. Int J Oral Maxillofac Implants. 2018 Jan/Feb;33(1):197-205.

**b. Table S1. Detailed data of the included studies**

| **Study** | **Year** | **Study Design** | **Country / Setting** | **Patients (male/ female) (n)** | **Patients’ age range (mean) (years)** | **Loading** | **Location (maxilla/ mandible)** | **Number of smokers (n)** | **Attachment system** |
| --- | --- | --- | --- | --- | --- | --- | --- | --- | --- |
| Ahn | 2004 | RS (unicenter) | South Korea / University | 11 (4/7) | 41-73 (52.9) | Immediate | Md | NA | O’Ring - Ball |
| Araujo | 2015 | Case report | Brazil / University | 1 (0/1) | 75 | Immediate | Md | 0 | O’Ring - Ball |
| Bellia | 2018 | RS (unicenter) | Italy / University | 11 (3/8) | 54-85 (68) | Immediate | Md | Yes, but exact number not informed | Locator |
| Bielemann | 2022 | PS  (unicenter) | Brazil / University | 16 (6/10) | NM (68.1) | Delayed (3 mo) | Md | 0 | Locator-like (Equator) |
| Brandt | 2012 | NA (unicenter) | USA / University | 24 (NA) | NA | Immediate | Md | 7 | O’Ring - Ball |
| Catalán | 2016 | NA (unicenter) | Chile / University | 7 (NA) | 62-74 (NA) | 2-3 wk | Md | NA | O’Ring – Ball |
| Chatrattanarak | 2022 | PS  (unicenter) | Thailand / University | 26 (NA) | 35-89 (70.1) | Immediate | Md | 0 | Locator-like (Equator) |
| Curado | 2023 | PS  (unicenter) | Brazil / University | 74 (26/48) | NA (64.1) | Immediate  Delayed (6 wk) | Md | 15  (21 former) | Locator-like (Optiloc) |
| de Souza | 2015 | PS  (unicenter) | Brazil / University | 80 (24/56) | NA (59) | Delayed (3 mo) | Md | Yes, but exact number not informed | O’Ring – Ball |
| Elsyad | 2016 | PS  (unicenter) | Egypt / University | 28 (16/12) | 49-75 (62.9) | Immediate | Md | 0 | O’Ring – Ball |
| Elsyad | 2019 | PS  (unicenter) | Egypt / University | 20 (NA) | 55-65 (59.2) | Immediate | Md | 0 | O’Ring - Ball |
| Enkling | 2020 | PS  (unicenter) | Switzerland / University | 20 (5/15) | 41-87 (66.5) | Immediate | Md | NA | O’Ring - Ball |
| Griffitts | 2005 | RS  (unicenter) | USA / Private practice | 24 (NA) | 50-90 (67) | Immediate | Md | NA | O’Ring - Ball |
| Hussein | 2020 | PS  (unicenter) | Saudi Arabia / University | 27 (27/0) | 45-60 (56) | Immediate | Md | 27 | O’Ring - Ball |
| Jawad | 2017 | PS  (unicenter) | UK / University | 22 (10/22) | 52-88 (68.5) | Delayed (2 mo) | Md | 0 | O’Ring - Ball |
| Jofré | 2010 | PS  (unicenter) | Chile / Public service | 45 (18/27) | NM (71) | Immediate | Md | 2 | Bar clip  O’Ring - Ball |
| Kabbua | 2020 | PS  (unicenter) | Thailand / University | 31 (21/10) | NM (65.8) | Immediate | Md | Yes, but exact number not informed | Locator-like (Equator) |
| Kämmerer | 2021 | PS  (unicenter) | Germany / University | 20 (6/14) | 56-87 (69.6) | Immediate  Delayed | Md | NA | O’Ring - Ball |
| Kilic | 2017 | Case report | Turkey / University | 1 (1/0) | 6 | Delayed (4 mo) | Md | 0 | O’Ring - Ball |
| Kovacic | 2018 | NA  (unicenter) | Croatia / University | 5 (1/4) | 72-82 (NA) | Delayed (6-8 wk) | Md | 0 | O’Ring - Ball |
| Kumari | 2016 | Case report | India / Public service | 1 (0/1) | 69 | Delayed (4 mo) | Md | NA | O’Ring - Ball |
| Maryod | 2014 | PS  (unicenter) | Egypt / University | 36 (20/16) | NA (64) | Immediate  Delayed (3 mo) | Md | 0 | O’Ring - Ball |
| Mifsud | 2020 | PS  (unicenter) | Malta / University | 25 (14/11) | NA (66.8) | Immediate | Md | Yes, but exact number not informed | Locator |
| Mundt | 2015 | RS (multicenter) | Germany / Private practice | 133 (54/79) | 48-100 (71.2) | Immediate  Delayed | Mx, Md | NA | O’Ring - Ball |
| Park | 2023 | RS  (unicenter) | South Korea / University | 30 (17/13) | 54-80 (70.7) | Immediate | Md | 0 | O’Ring - Ball |
| Possebon | 2021 | PS  (unicenter) | Brazil / University | 26 (8/18) | 50-90 (67.5) | Delayed (3 mo) | Md | Yes, but exact number not informed | Locator-like (Equator) |
| Preoteasa | 2014 | RS  (unicenter) | Romania / University | 23 (10/13) | 52-76 (62) | Delayed (3 mo) | Mx, Md | NA | O’Ring - Ball |
| Rujiraphan | 2021 | RS  (unicenter) | Thailand / University | 11 (5/6) | 54-92 (68) | Delayed (2 mo) | MX, Md | NA | Locator-like (Equator) |
| Scarano | 2012 | RS  (unicenter) | Italy / University | 38 (NA) | 60-92 (69) | Immediate | Md | NA | O’Ring - Ball |
| Scepanovic | 2012 | PS  (unicenter) | Serbia / University | 30 (14/16) | 45-63 (NA) | Immediate | Md | Yes, but exact number not informed | O’Ring - Ball |
| Schwindling | 2016 | RS (multicenter) | Germany, Luxemburg / Private practice | 25 (8/17) | 51-87 (72) | Immediate | Md | NA | O’Ring - Ball |
| Temizel | 2017 | PS  (unicenter) | Germany / University | 22 (NA) | NA | Delayed (4 mo) | Md | NA | O’Ring - Ball |
| Tomasi | 2013 | PS  (multicenter) | Sweden / University + Private practice | 21 (9/12) | 54-85 (71) | Immediate | Mx, Md | 3 | Ball (O’Ring was not used) |
| Topic | 2022 | PS  (multicenter) | Croatia / University + Private practice | 30 (10/20) | NA (65.1) | Early (7-10 days) | Md | Yes, but exact number not informed | O’Ring - Ball |
| Van Doorne | 2023 | PS (multicenter) | Belgium / University | 31 (17/14) | NA (62.3) | Delayed (6 mo) | Mx | Yes, but exact number not informed | O’Ring - Ball |
| Worni | 2020 | Case report | Switzerland / University | 1 (1/0) | 61 | Immediate | Mx, Md | 0 | Locator-like (Optiloc) |
| Yilmaz | 2020 | Case report | Switzerland / University | 1 (NA) | 68 | Delayed (4 mo) | Mx | 1 | Locator-like (Optiloc) |
| Zygogiannis | 2016 | PS (multicenter) | Netherlands / University + Private practice | 8 (6/2) | 58-79 (70.6) | Immediate | Md | 0 | O’Ring - Ball |
| Zygogiannis | 2017 | PS (multicenter) | Netherlands / University | 25 (13/12) | NA (67) | Immediate | Md | 0 | O’Ring - Ball |

NA – not available; PS – prospective study; RS – retrospective study

Mx – maxilla; Md - mandible

wk – weeks; mo – months

**c. Table S2. Quality assessment of the included studies, according to the National Institutes of Health (NIH)**

| Study | Year | Was the study question or objective clearly stated? | Was the study population clearly and fully described, including a case definition? | Were the cases consecutive? | Were the subjects comparable? | Was the intervention clearly described? | Were the outcome measures clearly defined, valid, reliable, and implemented consistently across all study participants? | Was the length of follow-up adequate? | Were the statistical methods well-described? | Were the results well-described? | Total (n/9) |
| --- | --- | --- | --- | --- | --- | --- | --- | --- | --- | --- | --- |
| Ahn | 2004 | 1 | 1 | 1 | 1 | 1 | 1 | 1 | 0 | 1 | 8/9 |
| Bellia | 2018 | 1 | 1 | 0 | 1 | 1 | 1 | 1 | 1 | 1 | 8/9 |
| Bielemann | 2022 | 1 | 1 | 0 | 1 | 1 | 1 | 1 | 1 | 1 | 8/9 |
| Brandt | 2012 | 1 | 1 | 0 | 1 | 1 | 1 | 1 | 1 | 0 | 7/9 |
| Catalán | 2016 | 1 | 1 | 0 | 1 | 1 | 1 | 1 | 1 | 1 | 8/9 |
| Chatrattanarak | 2022 | 1 | 1 | 0 | 1 | 1 | 1 | 1 | 1 | 1 | 8/9 |
| Curado | 2023 | 1 | 1 | 1 | 1 | 1 | 1 | 1 | 1 | 1 | 9/9 |
| de Souza | 2015 | 1 | 1 | 0 | 1 | 1 | 1 | 1 | 1 | 1 | 8/9 |
| Elsyad | 2016 | 1 | 1 | 0 | 1 | 1 | 1 | 1 | 0 | 1 | 7/9 |
| Elsyad | 2019 | 1 | 1 | 0 | 1 | 1 | 1 | 1 | 0 | 1 | 7/9 |
| Enkling | 2020 | 1 | 1 | 0 | 1 | 1 | 1 | 1 | 1 | 1 | 8/9 |
| Griffitts | 2005 | 1 | 1 | 1 | 1 | 1 | 1 | 1 | 0 | 0 | 7/9 |
| Hussein | 2020 | 1 | 1 | 0 | 1 | 1 | 1 | 1 | 1 | 1 | 8/9 |
| Jawad | 2017 | 1 | 1 | 0 | 1 | 1 | 1 | 1 | 1 | 1 | 8/9 |
| Jofré | 2010 | 1 | 1 | 0 | 1 | 1 | 1 | 1 | 0 | 1 | 7/9 |
| Kabbua | 2020 | 1 | 1 | 0 | 1 | 1 | 1 | 1 | 1 | 1 | 8/9 |
| Kämmerer | 2021 | 1 | 1 | 0 | 1 | 1 | 1 | 1 | 0 | 1 | 7/9 |
| Kovacic | 2018 | 1 | 1 | 1 | 1 | 1 | 1 | 1 | 1 | 1 | 9/9 |
| Maryod | 2014 | 1 | 1 | 0 | 1 | 1 | 1 | 1 | 1 | 1 | 8/9 |
| Mifsud | 2020 | 1 | 1 | 0 | 1 | 1 | 1 | 1 | 1 | 1 | 8/9 |
| Mundt | 2015 | 1 | 1 | 1 | 1 | 1 | 1 | 1 | 1 | 1 | 9/9 |
| Park | 2023 | 1 | 1 | 1 | 1 | 1 | 1 | 1 | 1 | 1 | 9/9 |
| Possebon | 2021 | 1 | 1 | 1 | 1 | 1 | 1 | 1 | 1 | 1 | 9/9 |
| Preoteasa | 2014 | 1 | 1 | 0 | 1 | 1 | 1 | 1 | 0 | 1 | 7/9 |
| Rujiraphan | 2021 | 1 | 1 | 1 | 1 | 1 | 1 | 1 | 0 | 0 | 7/9 |
| Scarano | 2012 | 1 | 1 | 1 | 1 | 1 | 1 | 1 | 0 | 0 | 7/9 |
| Scepanovic | 2012 | 1 | 1 | 1 | 1 | 1 | 1 | 1 | 1 | 1 | 9/9 |
| Schwindling | 2016 | 1 | 1 | 1 | 1 | 1 | 1 | 1 | 0 | 1 | 8/9 |
| Temizel | 2017 | 1 | 1 | 1 | 1 | 1 | 1 | 1 | 1 | 1 | 9/9 |
| Tomasi | 2013 | 1 | 1 | 0 | 1 | 1 | 1 | 1 | 1 | 1 | 8/9 |
| Topic | 2022 | 1 | 1 | 1 | 1 | 1 | 1 | 1 | 0 | 1 | 8/9 |
| Van Doorne | 2023 | 1 | 1 | 1 | 1 | 1 | 1 | 1 | 0 | 1 | 8/9 |
| Zygogiannis | 2016 | 1 | 1 | 1 | 1 | 1 | 1 | 1 | 1 | 1 | 9/9 |
| Zygogiannis | 2017 | 1 | 1 | 1 | 1 | 1 | 1 | 1 | 1 | 1 | 9/9 |

**d. Table S3. Quality assessment of the included case reports, according to the CARE guidelines**

| Study | Year | 1 | 2 | 3a | 3b | 3c | 3d | 4 | 5a | 5b | 5c | 5d | 6 | 7 | 8a | 8b | 8c | 8d | 9a | 9b | 9c | 10a | 10b | 10c | 10d | 11a | 11b | 11c | 11d | 12 | 13 | T |
| --- | --- | --- | --- | --- | --- | --- | --- | --- | --- | --- | --- | --- | --- | --- | --- | --- | --- | --- | --- | --- | --- | --- | --- | --- | --- | --- | --- | --- | --- | --- | --- | --- |
| Araújo | 2015 | 1 | 0 | 1 | 1 | 1 | 1 | 1 | 1 | 1 | 1 | 1 | 1 | 1 | 1 | - | - | - | 1 | - | - | 1 | 1 | - | 1 | 1 | 1 | 1 | 1 | 0 | 1 | 22 |
| Kilic | 2017 | 0 | 0 | 1 | 1 | 1 | 1 | 1 | 1 | 1 | 1 | 1 | 1 | 1 | 1 | - | - | - | 1 | - | - | 1 | 1 | - | 1 | 1 | 1 | 1 | 1 | 0 | 1 | 21 |
| Kumari | 2016 | 1 | 0 | 1 | 1 | 1 | 1 | 1 | 1 | 1 | 1 | 1 | 1 | 1 | 1 | - | - | - | 1 | - | - | 1 | 1 | - | 1 | 1 | 1 | 1 | 1 | 0 | 1 | 22 |
| Worni | 2020 | 0 | 0 | 1 | 1 | 1 | 1 | 1 | 1 | 1 | 1 | 1 | 1 | 1 | 1 | - | - | - | 1 | - | - | 1 | 1 | - | 1 | 1 | 1 | 1 | 1 | 0 | 1 | 21 |
| Yilmaz | 2020 | 0 | 0 | 1 | 0 | 0 | 0 | 1 | 1 | 1 | 1 | 1 | 1 | 1 | 1 | - | - | - | 1 | - | - | 1 | 1 | - | 1 | 1 | 1 | 1 | 1 | 0 | 1 | 18 |

Items 8b, 8c, 8d, 9b, 9c, and 10c are not applicable. Therefore, 24 out the 30 items are considered

T – total score (out of 24)
